# The macronuclear genome of the Antarctic psychrophilic marine ciliate *Euplotes focardii* reveals new insights on molecular cold adaptation

**DOI:** 10.1038/s41598-021-98168-5

**Published:** 2021-09-21

**Authors:** Matteo Mozzicafreddo, Sandra Pucciarelli, Estienne C. Swart, Angela Piersanti, Christiane Emmerich, Giovanna Migliorelli, Patrizia Ballarini, Cristina Miceli

**Affiliations:** 1 School of Biosciences and Veterinary Medicine, University of Camerino, 62032 Camerino, MC Italy; 2 Max Planck Institute for Developmental Biology, Tübingen, Germany

**Keywords:** Evolutionary ecology, Comparative genomics

## Abstract

The macronuclear (MAC) genomes of ciliates belonging to the genus *Euplotes* species are comprised of numerous small DNA molecules, nanochromosomes, each typically encoding a single gene. These genomes are responsible for all gene expression during vegetative cell growth. Here, we report the analysis of the MAC genome from the Antarctic psychrophile *Euplotes focardii.* Nanochromosomes containing bacterial sequences were not found, suggesting that phenomena of horizontal gene transfer did not occur recently, even though this ciliate species has a substantial associated bacterial consortium. As in other euplotid species, *E. focardii* MAC genes are characterized by a high frequency of translational frameshifting. Furthermore, in order to characterize differences that may be consequent to cold adaptation and defense to oxidative stress, the main constraints of the Antarctic marine microorganisms, we compared *E. focardii* MAC genome with those available from mesophilic *Euplotes* species*.* We focussed mainly on the comparison of tubulin, antioxidant enzymes and heat shock protein (HSP) 70 families, molecules which possess peculiar characteristic correlated with cold adaptation in *E. focardii*. We found that α-tubulin genes and those encoding SODs and CATs antioxidant enzymes are more numerous than in the mesophilic *Euplotes* species. Furthermore, the phylogenetic trees showed that these molecules are divergent in the Antarctic species. In contrast, there are fewer *hsp70* genes in *E. focardii* compared to mesophilic *Euplotes* and these genes do not respond to thermal stress but only to oxidative stress. Our results suggest that molecular adaptation to cold and oxidative stress in the Antarctic environment may not only be due to particular amino acid substitutions but also due to duplication and divergence of paralogous genes.

## Introduction

*Euplotes focardii* is an Antarctic ciliate classified as an obligate psychrophilic stenothermal organism^1–4^. As all ciliates, *E. focardii* is characterized by the presence of cilia on its surface and by nuclear dimorphism: a micronucleus (MIC) that represents the germ line, and a macronucleus (MAC) serving as the somatic line involved in the gene expression during the vegetative stage. The MAC derives from the MIC after extensive DNA rearrangements during conjugation, including the fragmentation of chromosomes and the elimination of non-protein-coding DNA segments^5^. As a consequence, the MAC genome is commonly composed by nanochromosomes, i.e., tiny chromosomes capped by telomeric sequences that, in general, contain a single coding sequence (CDS)^6,7^. The nanochromosomes are amplified to thousands of copies (~2000)^5^. The copy number may oscillate since it is probably unregulated through cell replication, as it has been described for *Stylonychia*^8^ and *Oxytricha*^9^.

As a psychrophilic unicellular organism directly exposed to environmental cues, *E. focardii* represents an excellent model for the study of cold adaptation, offering some potential advantages over psychrophilic multicellular models^10^. Low temperatures exert several physicochemical constraints on cold living organisms, including the process of microtubule polymerization^11^. Microtubule polymers are of fundamental importance in many eukaryotic cellular processes, including cell motility, maintenance of cytoskeletal architecture, intracellular transport, and mitosis. Microtubules assemble from α- and β-tubulin heterodimers with the help of γ-tubulins, a structural component of both centrioles and basal bodies. There are other components of the tubulin superfamily, such as the δ-tubulin and the ε-tubulin, that most likely interact longitudinally with α-tubulins at the minus ends and with β-tubulins to the plus ends of microtubules, respectively^12^. Furthermore, δ-tubulin may be involved in the formation of the C triplet tubules in the basal bodies, while ε-tubulin may be located to the centrosome^13^. Generally, in organisms that live in temperate environments, the assembly of microtubules from tubulin heterodimers requires physiological temperatures and these microtubules usually disassemble at temperatures below 4 °C. In chronically cold habitats, the microtubule dynamics in psychrophilic organisms, including the Antarctic ciliate *E. focardii,* most likely reflect adaptive modifications of tubulin heterodimers^11,14–16^.

*E. focardii* is also a good model for studying adaptation to oxidative stress. Like any Antarctic marine microorganism, this ciliate is constantly exposed to a high oxygen concentration and abundant reactive oxygen species (ROS), due to the higher O_2_ solubility in water at low temperature and to high UV radiation due to the ozone hole. Accordingly, strengthened defenses against oxidative stress, e.g., by increasing the antioxidant enzymes system, including superoxide dismutase (SOD), catalase (CAT), peroxiredoxins (PRX), thioredoxin reductase (TRXR) and glutathione systems (glutathione synthetase GS, glutathione reductase GR, glutathione peroxidases GPx, and glutathione S-transferases GST) may be necessary. Specifically, the dismutation by SOD of the superoxide (the primary produced Reactive Oxygen Species, abbreviated as ROS), and the reduction of the produced H_2_O_2_ by CAT and GPx (often in association with thiol-containing enzymes, PRX, TRXR and glutaredoxins^17^) are of extreme importance for stress response in all cells^18^. Likewise, GSTs are important to inactivate unsaturated aldehydes, epoxides, and hydroperoxides, secondary metabolites of the reactions described above^19^. In this regard, it was shown that the transcription regulation of SOD, CAT, GR and GPx is strongly affected by oxidative stress in the mesophilic ciliate *Tetrahymena thermophila*^20^*.*

Thermal stress response could also be a further biomarker to understand the adaptation of these organisms. In mesophilic organisms, heat shock proteins (HSPs) are the main macromolecules involved in this mechanism and, acting as chaperones, provide the stabilization, the partial refolding, or the detection of proteins irreversibly damaged^21,22^. In particular, the Hsp70 group is represented by proteins that are quickly induced under stress conditions by the activation of the *hsp70* gene expression^23^. However, *hsp70* genes have been reported to have lost heat inducibility in several Antarctic marine organisms, including *E. focardii*^24–26^.

*Euplotes* species are characterized by an unusual and pervasive mechanism of programmed translational frameshifting^27^. A previous study^28^ reported an extensive analysis of this mechanism in the *E. crassus* genome and included some comparison with the *E. focardii* genome to gain some insights into the conservation and molecular basis of this frameshifting. Here, we report a deeper *E. focardii* MAC genome analysis based on reads that have been completely reassembled and annotated. Furthermore, we focused on characterizing Hsp70, tubulin and antioxidant enzymes gene families. Our results suggest that molecular adaptation to cold and oxidative stress defense in the Antarctic environment may be based on a variable number of paralogous genes.

## Results and discussion

### A draft *E. focardii* macronuclear genome assembly

The *E. focardii* MAC genome assembly obtained using the SPAdes algorithm showed a significant improvement (25%) of the number of 2-telomere nanochromosomes with respect to the assembly previously produced by Newbler (stored in GenBank as version MJUV01000000)^28^, i.e., 17,798 sequences containing telomeres at both ends (Table 1) vs 12,922 previously reported. SPAdes assembly was performed on cleaned reads, e.g., after the clean-up of all algal or bacterial contaminants (see "Materials and methods" section). A summary of the SPAdes assembly data is shown in Table 1: these data were obtained after an extra clean-up of sequences without telomeres and with a high GC content (Fig. S1). The final GC distribution shows a well-defined peak (with respect to the distribution before cleaning) centered on the 31.51% (Table 1, Fig. S2), a value consistent with those reported for other ciliates with a nanochromosomal MAC genome architecture (i.e., *Stylonychia lemnae*, *Oxytricha trifallax*, and *Euplotes crassus*, 31.5%, 31.2% and 36.9%, respectively)^29^. This homogeneous normal GC distribution is consistent with negligible bacterial contamination. Although we cannot rule out the presence of sequences belonging to yet unreported endosymbionts with low GC content, it is unlikely their sequence base composition would perfectly match that of *E. focardii* and produce a unimodal distribution. Nanochromosomes containing bacterial sequences were not found in the final assembly suggesting that no phenomena of horizontal gene transfer recently occurred in this ciliate even though it has a substantial associated bacterial consortium^30^.

**Table 1:** *E. focardii* macronuclear genome assembly statistics in comparison with *E. vannus*^79^ and *E. octocarinatus*^38^.

	*E. focardii*	*E. vannus*	*E. octocarinatus*
Assembler name and version	SPAdes 3.10.1	SPAdes 3.7.1	Mira 4.0 / SPAdes 2.5.0
Assembly size (Mb)	49.33	85.1	88.9
%GC	31.51	37.0	28.2
Contigs (n)	31,114	38,245	41,980
Telomeres (n)	87,301	156,713	132,894
N50 (bp)	2047	2714	2947
Mean contig length (bp)	1583	2225	2117
Max contig length (bp)	50,409	40,045	53,269
2-telomeres contigs (n)	17,798	25,871	30,058
Mean 2-telomere contig length (bp)	1954	2369	2474
1-telomeres contigs (n)	6136	7637	4368
Mean 1-telomere contig length (bp)	1280	2039	2114
0-telomeres contigs (n)	7180	4737	7554
Mean 0-telomers contig length (bp)	927	1736	700
% Contigs (with telomere)	76.9	87.2	81.9

The genome size is in line with expectations based on the previous studies on other ciliates^5^, as well as for the ciliates reported in Table 2. This number of nanochromosomes was chosen as the main parameter for the selection of the best assembly among those produced by the different versions and configurations/parameters of SPAdes algorithm described in "Materials and methods". This Whole Genome Shotgun project has been deposited at DDBJ/ENA/GenBank under the accession MJUV00000000. The version described in this paper is version MJUV02000000.

**Table 2:** *E. focardii* Gene prediction features in comparison with *E. vannus*^79^ and *E. octocarinatus*^38^.

	*E. focardii*	*E. vannus*	*E. octocarinatus*
Nanochromosomes with only 1 gene (n)	23,224	/	/
Nanochromosomes with more genes (n)	2650	/	/
Total genes (n)	28,881	43,040	29,076
Exons (n)	81,715	175,735	96,843
Introns (n)	52,832	/	/
Average intron length (bp)	69.02	/	/
Average number of introns per gene (n)	1.83	/	/
Average CDS length (bp)	1085	1460	1178
Number of genes with assigned function	15,357 (53.17%)	/	/
Number of predicted enzymes	3306 (11.45%)	/	/

This assembly does not show any alternative fragmentation on the basis of the clustering analysis used in analyses of the *Oxytricha* MAC genome and consistent with previous observations in *E. crassus*^7,31^. Moreover, from the pairwise sequence identity analysis of the assembly (described in the "Paralog prediction" section of "Materials and methods"), *E. focardii*, and *E. octocarinatus*, do not show a peak at high (> 90%) sequence identity typical of heterozygous alleles, as instead *E. crassus* and *E. vannus* show, but only one peak of around 40% identity, likely due to the presence of a substantial number of paralogous genes (Fig. S3). The MAC genome assemblies of *E. crassus*, *E. octocarinatus* and *E. vannus* all have considerably more complete nanochromosomes than *E. focardii* (Table 1; *E. crassus* MAC genomes are unpublished but have in excess of 30,000 nanochromosomes). However, all three species also show an order of magnitude more paralogs around the 40% peak than *E. focardii*. The smaller number of complete nanochromosomes in *E. focardii* thus likely reflects a combination of three factors: (i) lower assembly contiguity; (ii) high genome homozygosity; (iii) fewer paralogs. In future further improvement of this genome would likely best be achieved by using long-read sequencing, such as that provided by Pacific Biosciences.

The results of the gene prediction performed using the AUGUSTUS software are summarized in Table 2. The number of genes (predicted in the nanochromosomes and in the contigs with no telomeres that blast with a database containing all ciliates available proteomes), the average of the CDSs’ length, of the introns’ length and of the number of introns per gene are consistent with those previously reported for *Stylonychia* and *Oxytricha*^7,32^, and for *E. crassus*^28,33^. Nanochromosomes encoding a single CDS represent 74.58% of the total, and nanochromosomes with more than one CDS are 8.51% of the total; these percentages are comparable to those of *Stylonychia* and *Oxytricha* (75% encoding a single CDS and higher than 7% with more than one CDS^7,32,34^).

To assess genome completeness, we compared the predicted proteins of the *E. focardii* macronuclear genome assembly with the CEG database: 93% of this database’s sequences (231) have homologs in the *E. focardii* assembly. This value may suggest a small amount of incompleteness of the genome assembly. However, 11 out of 17 of the sequences without matches are also absent from the O*xytricha* and *Stylonychia* genomes (Table 3), consistently with greater evolutionary distances of ciliates from the eukaryotes included in CEG, as also proposed for *Stylonychia*^7^. Genome annotation revealed a total of 90 ribosomal proteins, of which 36 belong to the standard eukaryotic small 40S subunit and 54 to the large 60S subunit. These values strongly support the present genome analysis, considering that the total standard eukaryotic ribosomal protein set that contains 32 proteins in the 40S subunit and 48 in the 60S subunit. Rfam analysis of structural RNAs in *E. focardii*, in addition to confirm the annotated tRNAs described in the next section, revealed the presence in the assembly of a nanochromosome (NODE_589) encoding both 18S and 28S rRNA genes, as well as 10 additional snRNA genes, including 3 for 5S rRNA and one for 5.8S rRNA. These results agree with those previously reported for *Oxytricha trifallax*^7^ and *Stylonychia lemnae*^32^. Taken together these results support the completeness of the *E. focardii* MAC genome assembly.

**Table 3:** CEGs missing from alignment results using default CEGMA criteria.

CEG name	*Stylonychia*	*Oxytricha*	*E.focardii*
DNA-directed RNA polymerase, subunit RPB10	KOG3497		KOG3497
Mitochondrial F1F0-ATP synthase, subunit delta/A TP16	KOG1758	KOG1758	KOG1758
6-Phosphogluconate dehydrogenase	KOG2653	KOG2653	KOG2653^#^
Sugar (pentulose and hexulose) kinases	KOG2531	KOG2531	KOG2531
Predicted snRNP core protein	KOG3448		
Glucose-6-phosphate 1-dehydrogenase	KOG0563	KOG0563	KOG0563
OTU (ovarian tumor)-like cysteine protease		KOG2606	
6-phosphogluconolactonase- like protein	KOG3147	KOG3147	KOG3147
Spindle assembly checkpoint protein	KOG3285	KOG3285	KOG3285
RNA polymerase II transcription initiation/nucleotide excision repair factor TFIIH, subunit SSL1	KOG2807		
UDP-glucose pyrophosphorylase	KOG2638	KOG2638	KOG2638
Mitochondrial import inner membrane translocase, subunit TIM13	KOG1733	KOG1733	KOG1733
Translation initiation factor 3, subunit g (eIF-3 g)	KOG0122	KOG0122	
Uncharacterized conserved protein	KOG2967		
Mitochondrial import inner membrane translocase, subunit TIM9		KOG3479	KOG3479
Predicted translation initiation factor related to eIF-2B alpha/beta/delta subunits (CIG2/IDI2)	KOG1468	KOG1468	KOG1468
Ubiquitin fusion-degradation protein		KOG1816	
Uncharacterized conserved protein		KOG3237	
Molecular chaperone Prefoldin, subunit 4		KOG1760	
Small nuclear ribonucleoprotein (snRNP) SMF		KOG3482	KOG3482*
60S ribosomal protein L38		KOG3499	KOG3499
60S ribosomal protein L39			KOG0002
Multifunctional methyltransferase subunit TRM112-like protein isoform 1			KOG1088
Retention in endoplasmic reticulum 1			KOG1688
Translationally-controlled tumor protein			KOG1727
Dolichyl-P-Glc:Man9GlcNAc2-PP-dolichyl glucosyltransferase			KOG2575
Ubiquitin-like protein 5			KOG3493*
H/ACA ribonucleoprotein complex subunit 3			KOG3503
Total CEGs not found	14	17	20 (17)

^#^Found into a contig without telomeres set (with %GC > 45).

*Found into the assembly set but not into the genes predicted set.

### Properties of *E. focardii* translation

Ciliates, including *Euplotes*, frequently use the standard stop codons in atypical ways compared to the usual eukaryote assignments^35,36^. As previously reported^28,37,38^, we detected abundant frameshifting motifs (AAA, AAT, ATA, AAC etc. codons followed by a stop codon, either TAA or TAG) in the *E. focardii* MAC genome (see "Materials and methods" section) consistent with pervasive + 1 programmed translational frameshifting in *Euplotes* ciliates. The frameshifting analysis on the predicted genes revealed that at least 4.2% of total genes could be affected by this phenomenon. This value could be underestimated since it excludes genes that had no BLAST hits (about 47%) to our sequence database and does not consider other possible frameshifting motifs beside those already identified in *E. crassus*^28^. Even though the comparison of the frameshifting sites between *E. focardii* and *E. crassus* revealed that these are not conserved in the same genes^28^, the occurrence of predicted frameshifting (just under 10%) would be in line with the observations in *E. crassus* by Klobutcher et al.^37^ and in *E. octocarinatus* by Wang et al*.*^38^. Considering the transcript-level quantity of these specific genes, as described in the "Materials and methods" section, the + 1 ribosomal frameshifting activity does not significantly affect the transcription in *E. focardii* on the basis of the statistical test used (*p*-value = 0.386).

61 tRNAs were predicted in the *E. focardii* MAC genome assembly. 55 unique tRNAs were encoded on nanochromosomes and appear to be sufficient for the translation of all codons. These tRNAs include one selenocysteine tRNA (encoded on NODE_51680), with the typical long variable arm characteristic of such tRNAs, and a putative paralogous pair of cysteine tRNA genes with TCA anticodons (tRNA-Cys(TCA)-1: NODE_55662; tRNA-Cys(TCA)-2: NODE_55665; 90% identical to each other), which resemble those previously reported for *E. crassus*^39^. The tRNA-Cys(TCA) paralogs are in turn paralogs of tRNA-Cys(GCA), as for *E. crassus*^39^.

**Figure 1:**
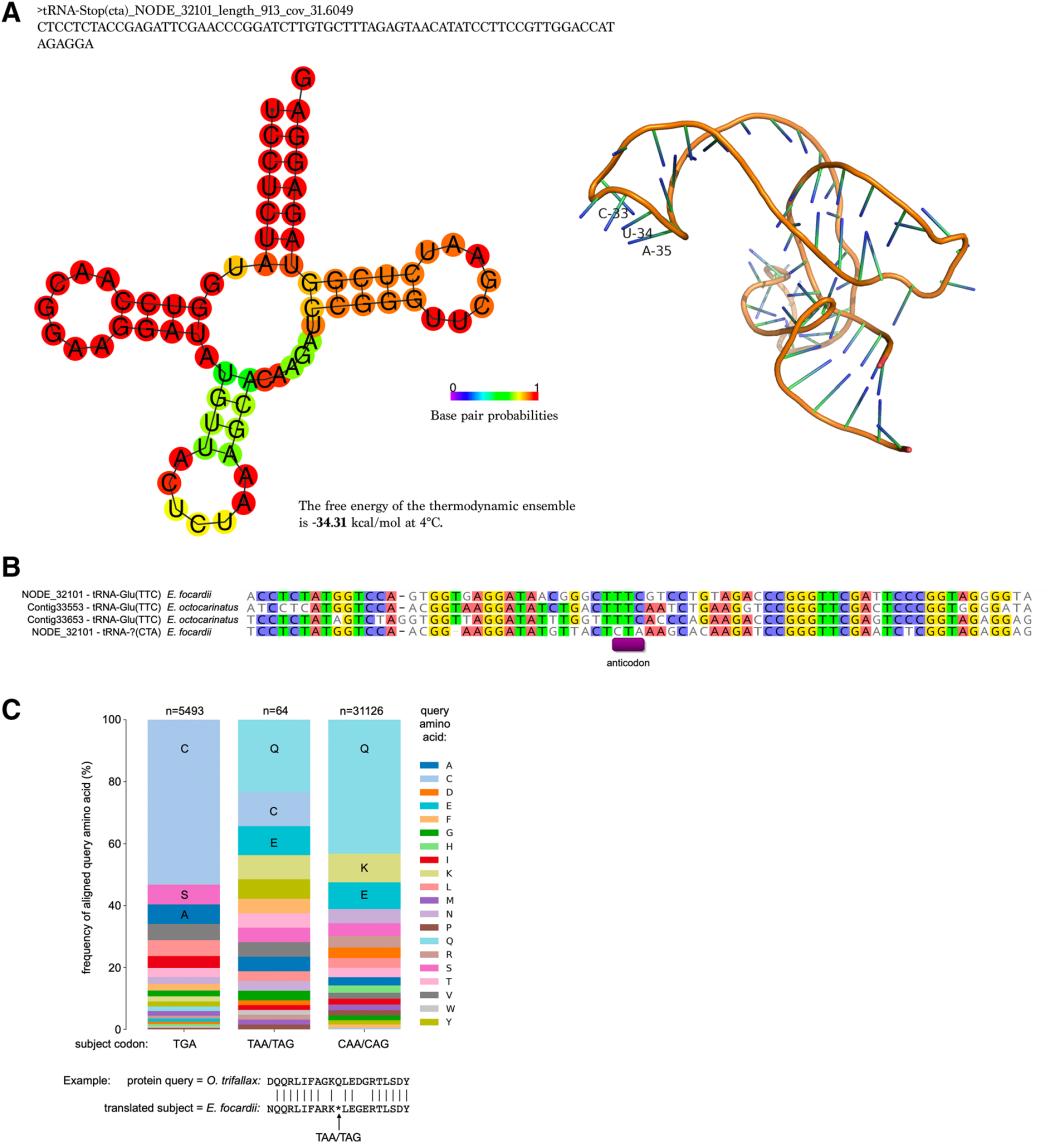
(**A**) Secondary and tertiary structures of a potential stop-suppressor tRNA of UAG. (**B**) alignment of predicted tRNA-Glu genes and candidate suppressor. (**C**) Frequencies of amino acids aligned to TGA = Cysteine, TAA/TAG and CAA/CAG = Glutamine in tblastn matches (amino acid counts were obtained from BLAST highest scoring pairs with e-value < 1e^−20^, a match criterion of 21 bp amino acids centered on the codon of interest and >  = 40% amino acid identity over 10 amino acids either side of subject codon – example provided below graph). “n” indicates the number of aligned codons analyzed for each bar. Query *O. trifallax* proteins were obtained from oxy.ciliate.org.

Previously a potential stop-suppressor tRNA of UAA, suggested to play a role in the ribosomal frameshifting was reported in *Euplotes octocarinatus*^38,40^. Though we found evidence of potential translational readthrough of stop codons (see subsequent analysis), we could not detect a similar tRNA in *Euplotes focardii* using BLASTN searches with the putative *E. octocarinatus* frameshifting tRNA as a query. Furthermore, neither tRNAscan-SE nor Aragorn predicted a similar tRNA to the putative frameshifting *E. octocarinatus* tRNA in *E. focardii*. The tRNA secondary structure prediction software did however predict an unrelated tRNA with a potential stop cognate anticodon on contig NODE_32101 (Fig. 1A). NODE_32101 encodes an additional tRNA-Glu(TTC), which is 66.7% identical to the putative suppressor tRNA(CTA) (Fig. 1). A different tRNA-Glu(TTC) encoded on a separate nanochromosome (NODE_54875) is 77% identical to NODE_32101 tRNA-Glu(TTC). BLASTN searches with both of the candidate tRNAs versus the *E. octocarinatus* genome yield top hits to *E. octocarinatus* Contig33653 (for NODE_32101 tRNA(CTA)) and Contig33553 (NODE_32101 tRNA-Glu(TTC)). For both *E. octocarinatus* nanochromosomes tRNA-Glu(TTC) genes are predicted in the regions of the BLASTN matches. Thus it appears NODE_32101 tRNA(CTA) is a paralog of Glu(TTC) tRNAs.

We observed expression of mature tRNAs for both predicted tRNAs on NODE_32101 in the YAMAT-seq reads (Supplemental Table 1), at low levels, but well within the limits of other predicted *E. focardii* tRNAs. It can be seen that the secondary structure predicted by RNAfold (Fig. 1A) does not place the anticodon 5’-CTA-3’ symmetrically in the anticodon loop, as is typically the case. However, this may be incorrect structure prediction, similar to the incorrect predictions by RNAfold we observed while attempting to predict the *E. focardii* tRNA-Cys(TCA) structures using this software. In the alignment of *E. focardii* and *E. octocarinatus* tRNAs, it can be seen that TCA aligns to the predicted TTC anticodons of the Glu-tRNAs (Fig. 1B). In future, to ascertain whether the candidate tRNA(CTA) on NODE_32101 is functional it would be necessary to search additional genomes from other *Euplotes* species, and observe both whether there are similar putative tRNA genes with TCA anticodons and also whether there are co-varying substitutions that support structural conservation and anticodon position.

Other than frameshifting “stop” codons, there are a few reports of potential translation of in-frame “stops”. In *E. focardii*, based on multiple sequence alignments, a TAG codon in a beta-tubulin gene was hypothesized to be translated as tryptophan^41^. Ricci et al*.* described the use of an in-frame TAG codon in two other *Euplotes* genes^42^. Recently, Wang et al*.* reported the use of TAG to encode an amino acid, likely glutamine, in the cathepsin B gene of the closely related freshwater *Euplotes octocarinatus*^43^. Given these observations and considerable plasticity in termination codon usage in ciliates^44,45^, we wondered if the *E. focardii* stop codons may occasionally be translated in other genes.

In standard genetic code organisms, which do not possess tRNAs directly cognate to “stop” codons, translational readthrough, may use near-cognate pairing of tRNAs (i.e., possessing two of the three complementary anticodon-codon pairings between bases). TAA/TAG codons are most frequently translated as glutamine and TGA as tryptophan in eukaryotic translational readthrough^46^. To investigate what amino acids TAA/TAG codons in *E. focardii* might encode, we examined the frequency of amino acids aligned to these codons in conserved alignments extracted from translated BLAST matches (Fig. 1C). We focused on matches with substantial sequence conservation up- and downstream of stops to exclude potential sites of translational frameshifting. As a baseline for comparison, we also examined the frequencies of amino acids aligned to TGA codons, which predominantly encode cysteine, and CAA/CAG codons, which encode glutamine. For both codon kinds, the most frequent aligned amino acids are the expected ones (Fig. 1C). TAA/TAG codons are most often frequently aligned to glutamine.

Whether TAA/TAG codons are translated by translational readthrough or conventionally by a tRNA such as the candidate tRNA(CTA) in *E. focardii*, remains to be determined. In other eukaryotes translational readthrough typically occurs at low levels, typically a small percentage of non-translational readthrough, and leads to short extensions of proteins^47^. Consequently, in future it will be of interest to determine the translational efficiency of in-frame stops in *Euplotes*, particularly in genes like the beta-tubulin paralog with an in-frame TAG occurring close to the N-terminus^41^, especially if it is a relatively highly translated protein like other beta-tubulins. Furthermore, it would also be of interest to determine what amino acid the candidate tRNA(CTA) may be charged with, and if there is transamidation of glutamate to glutamine, as observed in *Bacillus subtilis* and many other organisms^48,49^.

### The tubulin super families in *E. focardii* genome

The protein annotation procedure allowed the identification of 15,357 proteins (Table 2), 3306 of which are enzymes. Using CD-HIT, 5222 were grouped in clusters with at least 40% of identity. We focused on the analysis of the members of tubulins, antioxidant enzymes, and Hsp70 gene families to examine whether the number of paralogs and their evolution in each superfamily may be related to the *E. focardii* cold-adaptation.

In vitro polymerization studies performed with *E. focardii* purified tubulin heterodimers demonstrated their ability to form microtubules at temperatures close to the freezing point of the Antarctic marine habitat^14^, as also reported for tubulin heterodimers purified from Antarctic fishes^50,51^. In addition, the study of the tubulin superfamily in an Antarctic psychrophilic ciliate is even more interesting with respect to other psychrophilic organisms because it contributes not only to the understanding of the molecular basis of microtubule cold adaptation but also of microtubular structure complexity. Indeed, differently from other eukaryotic microorganisms, ciliates assemble 17 different types of microtubules throughout their life cycle ^52^, even though all microtubule functions are carried out in a single cell.

The macronuclear genome sequencing from several ciliates^7,32,53,54^ allowed the characterization of up to five alpha- and beta-tubulin isotypes. Therefore, a multigenic tubulin family is a common characteristic in ciliates and these tubulin isotypes may be responsible of the formation of functionally different microtubules^55^ and with different dynamics properties^14,16,56^.

In previous papers, we reported the characterization of a single α-tubulin^3^, four β-tubulin^1,14^ and two γ-tubulin isotypes^15^ from *E. focardii*. Previously, the presence of four β-tubulin isotypes induced us to hypothesize that in *E. focardii* microtubules cold adaptation was based mainly on molecular modification of the β-tubulin subunit of the heterodimer rather than on the α-tubulin subunit. The comparison of these sequences with the homologs from non-cold adapted *Euplotes* species revealed the presence of unique amino acid substitutions in the *E. focardii* tubulin isotypes that may be correlated with cold adaptation^14^. Therefore, we further investigated this relevant class of proteins in the *E. focardii* genome.

**Figure 2:**
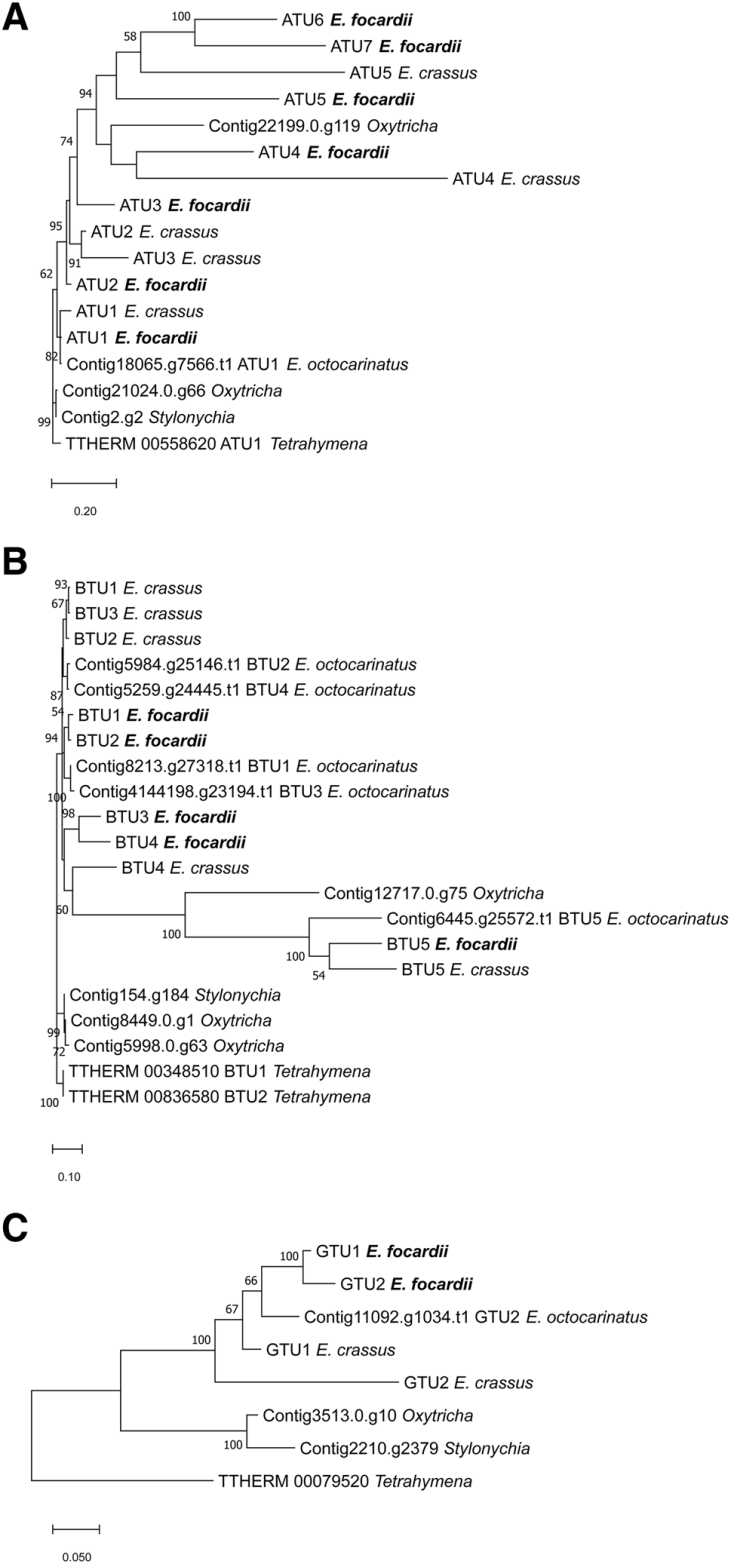
Phylogenetic trees of tubulin super families: (**A**) α-tubulins, (**B**) β-tubulins and (**C**) γ-tubulins. Only bootstrap percentage values higher than 50%, indicated near nodes, are shown.

All tubulin sequences that were detected in the final assembly were checked starting from those previously characterized^1,3,14,15,56^. All the sequences already collected into the UniProt Knowledgebase (TBβ1—UniProt: Q9N2N6; TBβ2—UniProt: C0L7F0; TBβ3—UniProt: C0L7F1; TBα1—UniProt: Q8WRT6; TBγ1—UniProt: A3F2R1; TBγ2—UniProt: A3F2R2) were identified and confirmed as reported in Table 4, with the exception of TBβ4 (the sequence collected as C0L7F2 in UniProt is included in the phylogenetic tree of Fig. 2B). However, new isotypes were discovered after protein annotation. Specifically, a new β-tubulin isotype (named TBβ5) and six additional α-tubulin isotypes. The relationship among these isotypes and those for other *Euplotes* species are shown in the phylogenetic trees in Fig. 2. In the trees, we introduced homologs from *Tetrahymena*, *Oxytricha* and *Stylonychia* to better evidence the degree of *E. focardii* gene amplification and divergency. *E. focardii* α-tubulins branches (highlighted as bold in Fig. 2A) are scattered throughout the phylogeny of ciliate homologs: ATU1 and ATU2 isotypes cluster with the homologs from *E. crassus* in the same group containing the “canonical” *Tetrahymena* α-tubulin, whereas ATU3 to ATU7 isotypes form a separate clade with *E. crassus* ATU4 and ATU5, and one isotype from *Oxytricha*. This result suggests that the isotypes from ATU1 to ATU5 preceded the divergence of *E. focardii* from the other *Euplotes* species, and were maintained in the Antarctic ciliate, whereas ATU6 and ATU7 derived from an additional event of gene duplication in *E. focardii* that gave origin to new distinct isotypes. An high number of isotypes may be considered as an additional strategy for tubulin cold adaptation beside the presence of unique residues substitutions in the primary structure of tubulin heterodimers^1^.

**Table 4:** Tubulins comparison between *E. focardii*, *E. crassus* and *E. octocarinatus*. *NODE_87922_length_309_cov_44.0984 (fragment of 309 nt without telomeres only present in the total non-cleaned assembly).

Tubulin	Protein assembly code	*E. crassus*	*E. octocarinatus*
α1	Protein_13754°	Protein_27015°	18,065.g7566.t1° ^§^
α2	Protein_13962 (94.88% of identity vs α1)	Protein_27022°	
α3	Protein_14463 (85%)	Protein_28219°	
α4	Protein_14635 (59%)	Protein_29453	
α5	Protein_14449 (57%)	Protein_30084	
α6	Protein_15234 (57%)		
α7	Protein_13592 (54%)		
β1	Protein_13059°	Protein_28250	8213.g27318.t1°
β2	Protein_14529°	Protein_23030	5984.g25146.t1° ^§^
β3	Protein_14159°	Protein_27936°	4,144,198.g23194.t1
β4		Protein_28002	5259.g24445.t1
β5	Protein_13114	Protein_20290	6445.g25572.t1
γ1	Protein_13695°	Protein_26311°	
γ2	* °	Protein_25799°	11,092.g1034.t1°
δ1	Protein_16384		24,288.g13101.t1
δ2	Protein_06776		30,256.g19015.t1
ε	Protein_13848		17,307.g6847.t1
			2182.g10828.t1
			29,123.g17894.t1
			29,737.g18500.t1
			414,497.g23602.t1
α-like	Protein_09756 (similar to Protein_13962) Protein_11578 (similar to Protein_15234) Protein_12280 (similar to Protein_15234) Protein_12357 (similar to Protein_13962) Protein_13335 (similar to Protein_15234) Protein_14210 (similar to Protein_15234 frag) Protein_16674 (similar to Protein_13754)		6 proteins
β-like	Protein_11303 (similar to Protein_14529) Protein_15589 (similar to Protein_14159)		6 proteins

Protein sequence already deposited in UniProtKB (the ID numbers of *E. crassus* proteins are: Q8MM87 for TBα1, Q8MU38 for TBα2, Q8MU39 for TBα3, P20365 for TBβ3, P54403 for TBγ1 and P54404 for TBγ2; the ID numbers of *E. octocarinatus* proteins are: Q08114 for TBα1, Q08115 for TBβ1, Q0GGY3 for TBβ2 and P90548 for TBγ2).

^§^Contig contains a further sequence portion (in comparison with that deposited in UniProtKB) most likely derived by an assembling or gene prediction error.

By contrast, *E. focardii* β-tubulin family appears less amplified and divergent than alpha tubulin. The branches (highlighted as bold in Fig. 2B) are less scattered and cluster with the corresponding *E. crassus* and *E. octocarinatus* homologs, even though BTU5 forms a well separated clade. BTU1 and BTU2, and BTU3 and BTU4, may be originated by recent gene duplications in *E.focardii.*

With the high number of different α-tubulin isotypes in the Antarctic ciliate *E. focardii*, we reconsidered the importance of the α-tubulin subunit in microtubule cold adaptation.

**Figure 3:**
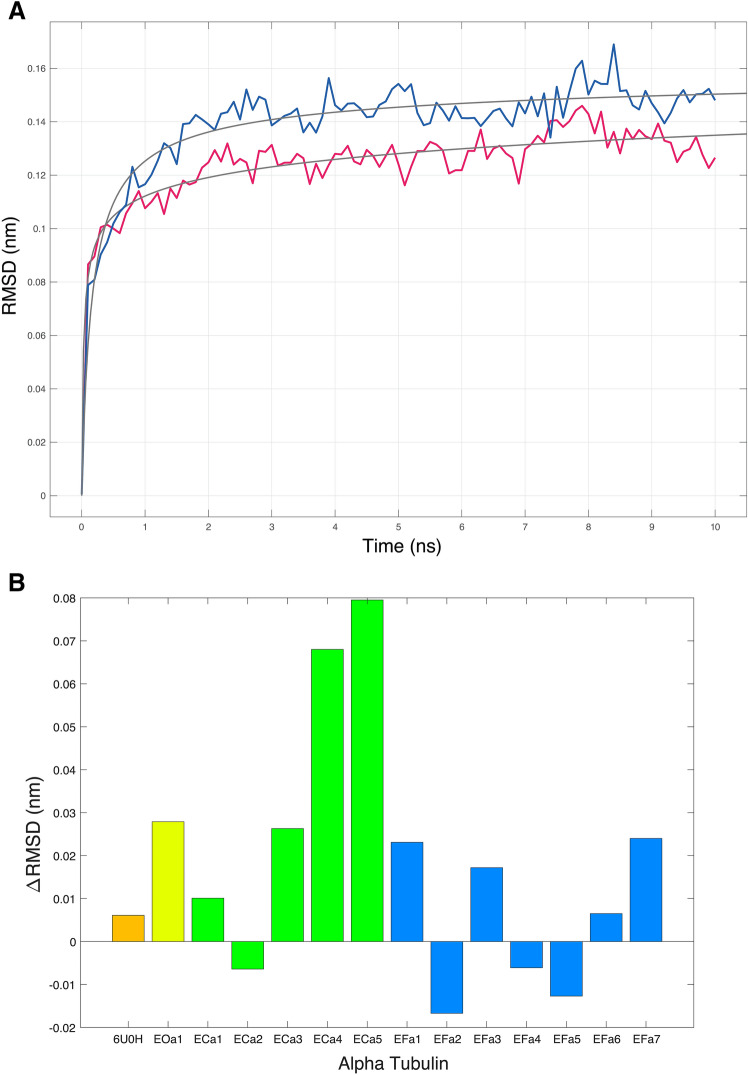
(**A**) trajectories data as backbone RMSDs for *E. focardii* α-tubulin isotype 2, for the first 10 ns, at 27 °C (red) and at 4 °C (blue). (**B**) the bar plot shows *ΔRMSD* values for each α-tubulin of *E. focardii* (blue), *E. crassus* (green), *E. octocarinatus* (yellow) and *T. thermophila* (6U0H, orange; ATU1 in Fig. 2) calculated as the difference between the *RMSD*_*max*_ at 27 °C and the *RMSD*_*max*_ at 4 °C (see "Materials and methods" section).

Molecular flexibility is regarded as a hallmark of cold adapted molecules, in particular enzymes, to cope with the reduction of dynamics and activity at low temperature^57,58^. We applied Molecular Dynamics (MD) simulation on each α-tubulin isotype of the ciliates under study. We found that only three *E. focardii* isotypes (2, 4 and 5; Fig. 3B) show a higher flexibility at 4 °C with respect to the *E. crassus* α-tubulin isotypes. The only exception in *E. crassus* is the isotype 2. This result can be considered as a further evidence of the *E. focardii* tubulin cold adaptation and suggests that not all isotypes must be flexible to function at low temperatures, as we reported also for the β-tubulin isotypes^14^. The similarity of *E. focardii* α-tubulin isotype 2 with that from *E. crassus* suggests a common origin of this α-isotype.

We also identified seven α-like and two β-like tubulins (e.g., more divergent isotypes; Table 4) and two δ and one ε isotypes. By contrast, the assembler was not able to produce two distinct complete contigs/nanochromosomes for γ-tubulin. In other words, γ-tubulin type 2 previously obtained from macronuclear DNA purified from *E. focardii* cells is present in the contig only in a short fragment form (see Table 4 legend). This is probably due to the high sequence similarity between the two γ-tubulin isotypes since raw reads specific for this second isotype were obtained after sequencing but difficult to align with this assembler. In the phylogenetic tree, *E. focardii* GTU1 and GTU2 isotypes (highlighted as bold in Fig. 2C) appear to be more related to the single γ-tubulin isotype from *E. octocarinatus* (that it is encoded by two similar genes) than the two distinct isotypes from *E. crassus*. In *E. focardii*, the presence of distinct γ-tubulin isotypes is associated to different roles in the nucleation of cellular microtubules (as previously reported^15^) more than to cold-adaptation.

### The antioxidant enzymes system

A major issue for Antarctic marine organisms is oxidative stress since they experience high dissolved oxygen, more soluble in cold seawaters, typical of Antarctic marine environment^59^. Two key classes of enzymes involved in antioxidant defenses are the superoxide dismutases and catalases.

Superoxide dismutases (SOD, EC 1.15.1.1) are the ubiquitous metalloenzymes that catalyse the dismutation of superoxide anion into molecular oxygen and hydrogen peroxide (O_2_^−^  + O_2_^−^  + 2H^+^  → O_2_ + H_2_O_2_)^60,61^. SODs are grouped into three protein families, based on the metal cofactor they contain and on the protein fold^62^. Copper, zinc SODs (Cu,Zn SODs) are found in the cytoplasm of eukaryotes, in the chloroplasts of some plants and in the periplasmic space of bacteria^63,64^. This group of SODs is often referred to as SOD1, but if these Cu,Zn enzymes are present in the extracellular fluids of eukaryotes are referred as SOD3^65,66^. Iron- and manganese-containing SODs (FeSOD and MnSOD, referred as SOD2) are considered the primitive forms of SODs^67,68^. FeSODs are found in prokaryotes and chloroplasts, while MnSODs are present both in prokaryotes and in the mitochondrial matrix of eukaryotes. Differently from MnSOD, Cu,Zn SODs were believed to be absent in protists^62^ until whole genomes sequencing revealed Cu,Zn SOD encoding genes in a number of different protists^69–72^.

In a previous paper^73^, two *E. focardii* Cu,ZnSODs and one MnSOD were biochemically characterized. All three SODs are active at 4 °C but at the same time they retain high activity upon 20 min incubation up to 55/60 °C. This feature is unusual in cold active enzymes that are often heat sensitive and undergo inactivation and unfolding even at mild temperature. Nevertheless, thermo-tolerance or even thermostability of cold adapted enzymes was previously reported^4,74^ suggesting that cold activity and thermo-tolerance may coexist in a molecule.

**Figure 4:**
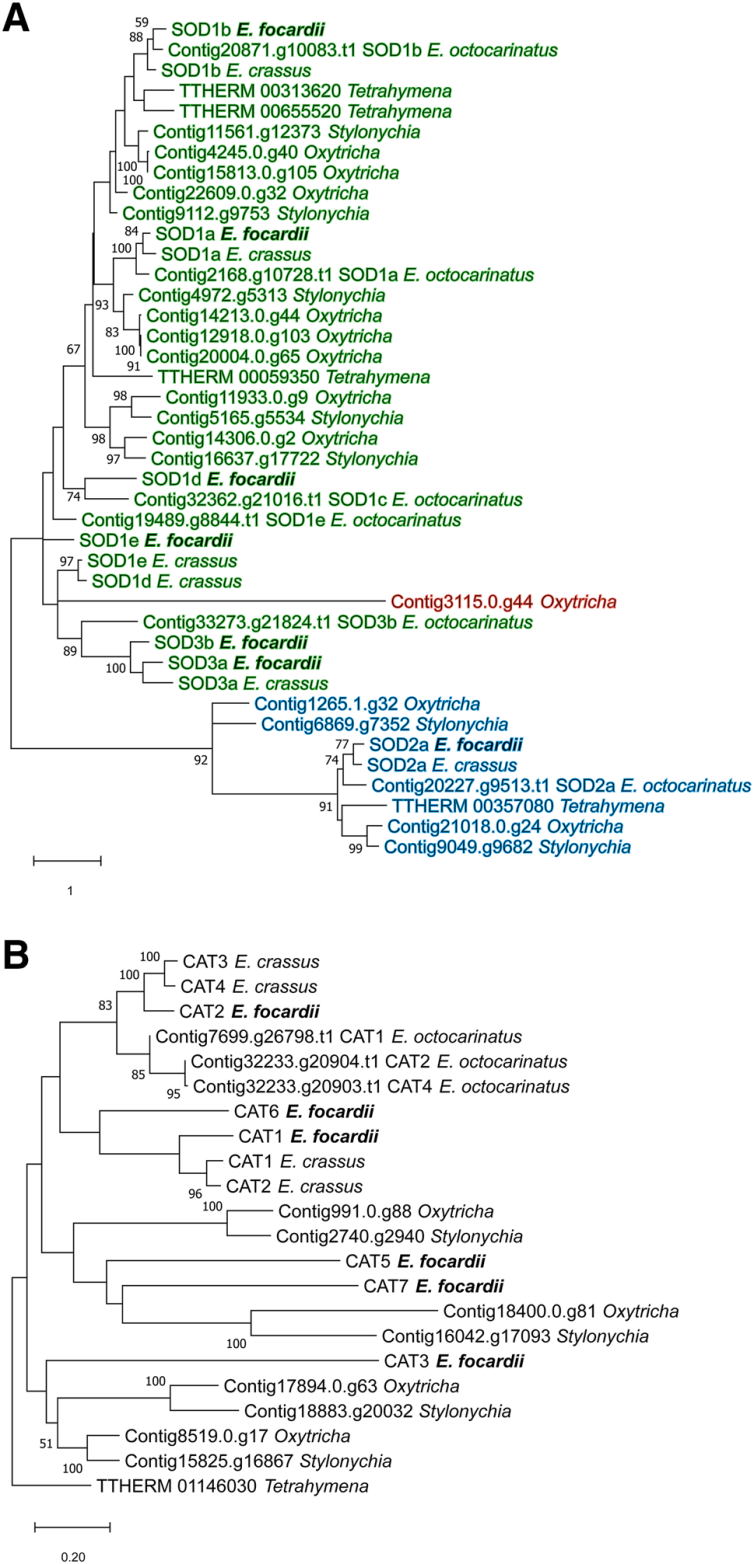
Phylogenetic trees of SODs (**A**) and CATs (**B**). Only bootstrap percentage values higher than 50%, indicated near nodes, are shown. Copper/zinc SODs are highlighted in green, iron/manganese SODs in blue, and nickel SODs in red.

The sequences of the enzymes previously studied and present in the UniProt Knowledgebase (SOD1a—UniProt: W0FZ77; SOD1b—UniProt: W0FUJ3; SOD2a—UniProt: MG575644) were confirmed by the analysis of the genes obtained in the *E. focardii* genome here described (Table 5): the SOD classification was done on the base of similarity with cytoplasmic SODs (type 1) and with mitochondrial SODs (type 2) and was confirmed by the presence of Cu/Zn and Fe/Mn pattern signature, respectively. In the same genome analysis, we could predict four additional *E. focardii* isoforms, the SOD1d and SOD1e and two SOD3 isoforms (Fig. 4A). According to this tree, the SOD3 isoforms appear to derive from gene duplication events probably happened before the divergence of *E. focardii* from other *Euplotes* species and some isotypes were then lost in *E. crassus*. As general result, the *E. focardii* SOD encoding gene family appeared composed by a higher number of genes with respect to the mesophilic *E. crassus*, probably due to a repeated event of gene duplication.

**Table 5:** Antioxidant enzyme comparison between *E. focardii*, *E. crassus* and *E. octocarinatus*.

Anti-oxidant enzyme	Protein assembly code	*E. crassus*	*E. octocarinatus*
SOD1a	Protein_25445 ^§^ °	Protein_49381 ^+^	2168.g10728.t1 ^§^
SOD1b	Protein_25349 ^§^ °	Protein_49686 ^§^	20,871.g10083.t1 ^§^
SOD1c			32,362.g21016.t1 ^+^
SOD1d	Protein_26998 ^+^	Protein_46068 ^§^	
SOD1e	Protein_23236 ^§^	Protein_46028 ^§^	19,489.g8844.t1 ^+^
SOD2a	Protein_23056 ^#^ °	Protein_44659 ^#^	20,227.g9513.t1 ^#^
SOD3a	Protein_23639 ^+^	Protein_44373 ^+^	
SOD3b	Protein_23528 ^+^		33,273.g21824.t1 ^+^
CAT1	Protein_12515	Protein_24239	7699.g26798.t1
CAT2	Protein_12995	Protein_24753	32,233.g20904.t1
CAT3	Protein_25541	Protein_24714	
CAT4		Protein_25374	32,233.g20903.t1
CAT5	Protein_27502		
CAT6	Protein_28050		
CAT7	Protein_23587		
PRX1	Protein_22541	Protein_45540	7627.g26732.t1
PRX2	Protein_22695	Protein_40156	7810.g26912.t1
PRX3	Protein_23342	Protein_45912	8218.g27323.t1
PRX4	Protein_23707	Protein_44467	6416.g25544.t1
PRX5a	Protein_28419		
PRX5b		Protein_44989	
PRX6		Protein_39913	
TRXR1	Protein_09887	Protein_21154°	9 proteins
TRXR2		Protein_20916°	
TRXR3	Protein_10344	Protein_19652	
GR1	Protein_14684	Protein_27646°	
GR2	Protein_14104	Protein_25768	
GPx1	Protein_21251	Protein_44685°	11,975.g1890.t1
GPx2	Protein_22198	Protein_44915°	13,463.g3154.t1
GPx3	Protein_23095	Protein_44916°	32,168.g20842.t1
GPx4	Protein_25138	Protein_43343	
GPx5	Protein_22265	Protein_44948	8754.g27852.t1
GPx6		Protein_48111	
GPx7		Protein_43209	
GS1	Protein_14094	Protein_25486	9010.g28082.t1
GS2	Protein_14269	Protein_37527	
GS3	Protein_14270	Protein_27010	
GST	69 proteins	60 proteins	63 proteins

°Protein sequence already deposited in UniProtKB (the ID numbers of *E.crassus* proteins are: B8XTW3 for TRXR1, B8XTW4 for TRXR2, J9SMC8 for GR1, B8XTW9 for GPx1, B8XTX0 for GPx2 and J9T5F4 for GPx3).

^§^Protein having Cu/Zn superoxide dismutase signature (Prosite pattern ID: PS00087 and PS00332).

^#^Fe/Mn superoxide dismutase signature (Prosite pattern ID: PS00088).

^+^Protein annotated as Cu/Zn superoxide dismutase but not have any known signature.

A similar situation is evident for the catalase (CAT) genes (Fig. 4B). CAT (EC 1.11.1.6), that inhibits the DNA damage by decomposing the H_2_O_2_ into oxygen and water induced by nitrofurazone, was previously considered a good biomarker for detecting oxidative stress and, consequently, ecotoxicity in aquatic ecosystems^75^. The number of *E. focardii* CATs genes is higher compared to the mesophilic *E. crassus*, probably due to a repeated event of gene duplication. As shown in Fig. 4B, CAT branches are scattered throughout the phylogeny of ciliate homologs. The low bootstrap values at these branches suggest that in *E. focardii* the CAT gene family underwent several events of recent gene duplications with potential adaptive outcomes that imply high divergence and consequently less supported phylogeny.

In general, the antioxidant enzymes system appears amplified in *E. focardii* (Table 6) suggesting that gene amplification may have contributed to combating the effects of increased oxygen concentration in the Antarctic seawaters. On the other hand, *E. focardii* possesses few gene encoding Thioredoxin NADPH Reductase (TrxR) and Glutathione Reductase (GR) isoforms. With the exception of *E. octocarinatus*, the genes for these enzymes are present in small number in the ciliate genomes known so far (see Table 6): *Tetrahymena*, as an exception in ciliates, has no TRXRs or GRs genes but 6 isotypes of thioredoxin–glutathione reductase (TGR) genes, that are composed by a fusion of the sequences of TRXR and glutaredoxin domains and are capable of transporting electrons from NADPH to both Trx and GSH systems^76^. *E. octocarinatus* has only 3 isotypes of TGR. In conclusion, the *E. focardii* antioxidant system appears to be based mainly on numerous SODs and CATs enzymes.

**Table 6:** Number of antioxidant enzymes predicted in ciliate organisms phylogenetically close to *E. focardii*. These values were obtained consulting the annotated proteins files.

	SOD	CAT	PRX	TRXR	GR	GPx	GS	GST	TGR	Total
*Euplotes focardii*	7	6	5	2	2	5	3	69	0	99
*Euplotes crassus*	6	4	6	3	2	7	3	60	0	91
*Euplotes vannus*	6	5	4	2	2	3	0	31	0	53
*Euplotes octocarinatus*	6	3	4	9	0	4	1	63	3	93
*Oxytricha trifallax*	11	4	0	1	0	10	2	58	0	86
*Stylonychia lemnae*	7	4	1	1	1	11	1	23	0	49
*Tetrahymena thermophila*	4	1	0	0	0	12	1	75	6	99

### The *E. focardii* heat-shock protein 70 gene family

**Figure 5:**
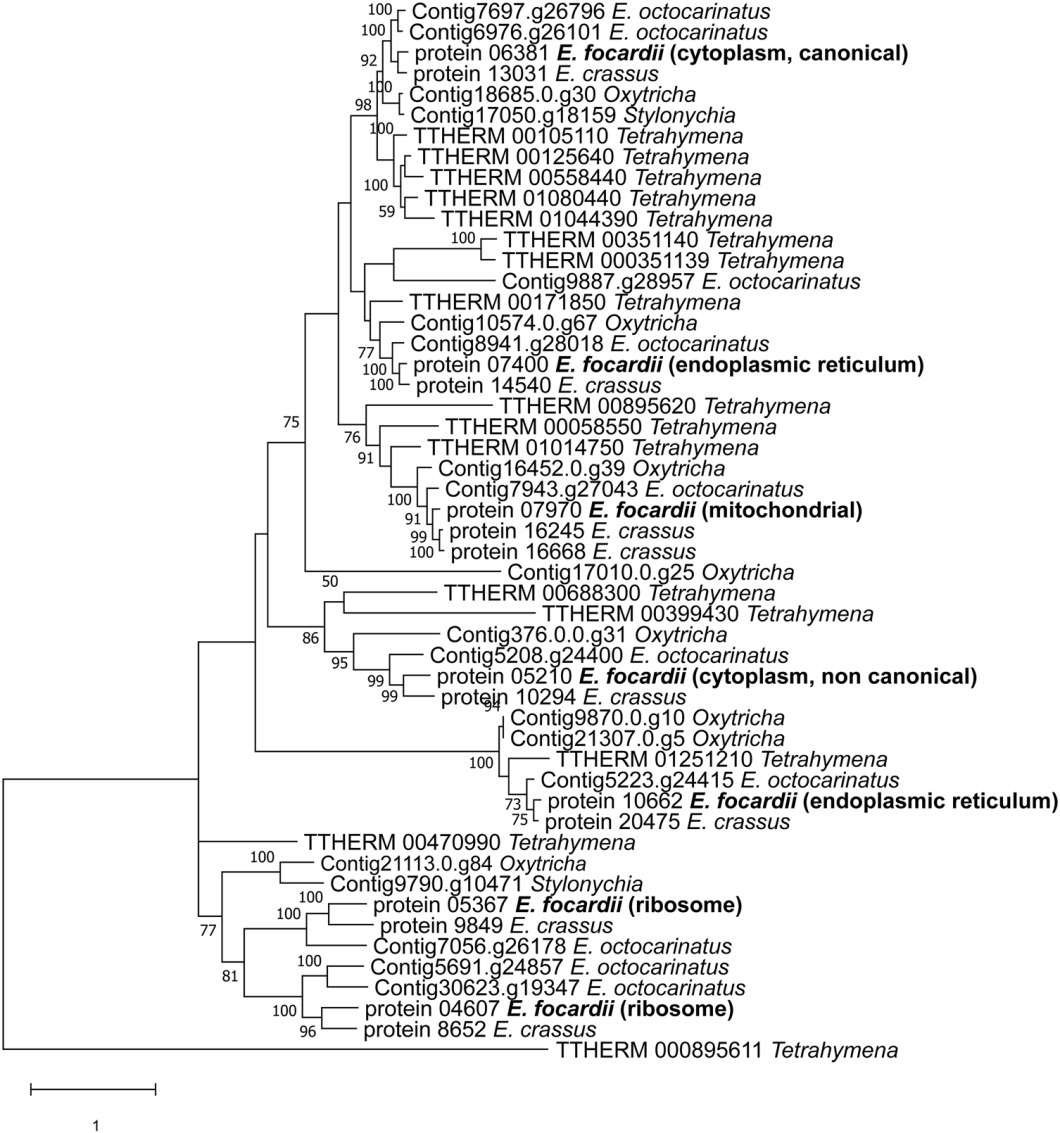
Phylogenetic tree of heat-shock protein Hsp70 super families. Only bootstrap percentage values higher than 50%, indicated near nodes, are shown.

The *E. focardii* macronuclear genome possesses seven distinct nanochromosomes that encode Hsp70 isoforms. According to their predicted C-terminal domain sequences^77^, we identified the respective Hsp70 subfamilies defined by the putative subcellular localization (Fig. 5 and Table SII). Figure 5 shows the phylogenetic relationships of the *Euplotes focardii* Hsp70 isoforms to orthologs from the mesophilic *Euplotes* species *E. crassus* and *E. octocarinatus* and from *O. trifallax*, *T. thermophila*, and *S. lemnae*. The topology of the tree supported sister relationships of all *E. focardii* Hsp70s with the orthologs from *E. crassus*, that represents the closest mesophilic *Euplotes*. Furthermore, our tree showed that all the *E. focardii* isoforms are of equal or even lower number than the corresponding orthologous from the other ciliates, indicating that in *E. focardii* genome there is no amplification of the *hsp70* encoding genes.

**Figure 6:**
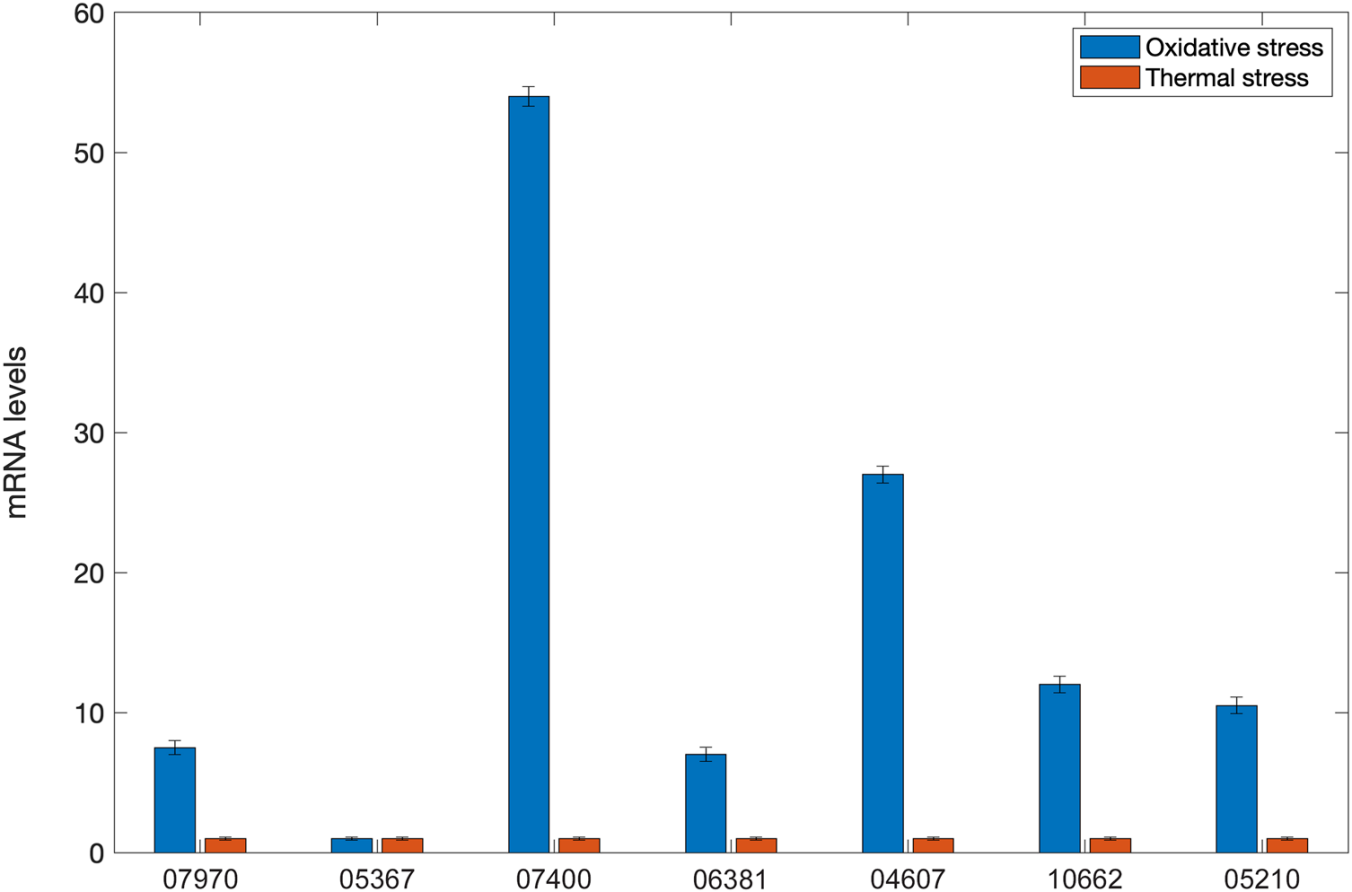
*Hsp70* transcript abundance in stressed *E. focardii* cells normalized to the abundance in control cells. mRNA levels were determined by qPCR of the Hsp70 isoforms (reported as protein assembly code) using the comparative threshold method. Mean ± SD (n = 4). Total RNA was purified from cells grown at their standard temperature of 4° C (i.e., not shocked), from cells which were exposed to 18 °C (orange) via a heat ramp (see "Materials and methods" section), or from cells exposed to 100 µM of hydrogen peroxide (blue); control and experimental treatments were for 30 min. Data are reported as the mean of three experiments.

La Terza et al.^26^ demonstrated that the canonical cytoplasmic *E. focardii hsp70* gene (GenBank acc. no. AAP51165.1, Protein_06381 in this paper) is not inducible by acute thermal stress, in contrast to the orthologous gene in *E. nobili* also found in Antarctica. Unlike in the Antarctic fish *Trematomus bernacchii*^25^, the *hsp70* gene does respond to several oxidative stressors, such as hydrogen peroxide in *E. focardii*^78^. In order to understand whether the *Euplotes* Hsp70 isoforms newly described here respond to thermal and/or oxidative stressors, we tested the inducibility of these genes in cultures subjected to heat shock (18 °C) or oxidative stress (H_2_O_2_). Figure 6 shows that thermal stress did not induce transcription of the any of the seven *Hsp70* genes relative to the control temperature (4 °C). In contrast, six of the seven genes (excluding isoform named Protein_05367) were induced by oxidative stress, strikingly so in the case of Protein_07400 isoform. We suggest that *E. focardii*, like several other Antarctic organisms, maintained a constitutive synthesis of Hsp70 isoforms to preserve protein function in the cold environment and evolved an oxidative stress response involving inducible Hsp70 synthesis. Moreover, this Antarctic ciliate has clearly lost the capability to induce the classical heat shock response when confronted with elevated temperatures.

## Conclusions

The Antarctic ciliate *E. focardii* represents an optimal model for studying genome adaptation to cold environments. In this paper, we reported the analysis of the *E. focardii* MAC genome after a significant improvement of the assembly that were also cleaned-up of algal or bacterial contaminants. In particular, we obtained 17,798 sequences containing telomeres at both ends vs 12,922 previously reported^28^. Even though some assembly parameters remain lower in comparison to the other ciliates reported, complete nanochromosomes are now closer, as a percentage of the total assembly, to those from other *Euplotes* species^38,79^ . This new report on the *E. focardii* MAC genome will provide additional information to investigate translation mechanisms in organisms with alternative genetic codes associated with the evolution of novel tRNA variants, including a putative suppressor tRNA, and to investigate how cold adaptation may have evolved.

The analysis of this improved *E. focardii* genome assembly allowed a better characterization of gene families, in particular that of the tubulins, that were previously only partially identified by single gene cloning approaches^1,3^. We identified a new β-tubulin isotype (TBβ5) and six additional divergent α-tubulin isotypes. In combination with the β-tubulin diversity, the role of the high number of different α-tubulins in microtubules cold adaptation should be reconsidered. Furthermore, we found that SODs and CATs families are composed by a higher number of genes with respect to the mesophilic *Euplotes*. The opposite trend was observed for the *hsp70* genes: in this case isoform’s diversification appears reduced with respect to homologs from other *Euplotes* species. Furthermore, expression of these genes was not induced by heat stress (18 °C for 30 min vs. a physiological temperature of 4 °C). On the other hand, hsp70 expression was raised during oxidative stress. We can conclude from these results that as for other Antarctic organisms, it is more important for *E. focardii* to cope with cold denaturation of proteins and oxidative stress than to respond to thermal stress. Consequently, the Hsp70 gene family did not expand like SODs and CATs families, that are involved in the antioxidant responses.

All these results suggest potential roles for paralogy in environmental adaptation, warranting future experimental investigation. Genomic expansions of specific protein gene families contributing to physiological fitness in freezing polar conditions have previously been reported for Antarctic notothenioids^80^. Gene diversification has been proved to produce a differential gene expression in specific adaptive conditions, as reported for the cold acclimation of the tea plant *Camellia sinensis*^81^ and also for the *E. focardii* βT3-tubulin during cilia regeneration^14^ and the *E. focardii* SOD 1b during cold stress^73^. In future, RNA-seq analyses of *E. focardii* transcriptome in different environmental conditions coupled to detailed molecular analyses will provide deeper insights into the role of duplicated genes.

In conclusion, we propose that the molecular basis of cold adaptation that enabled *E. focardii* to thrive in the Antarctic Ocean may not be solely due to particular amino acid substitutions that enable these molecules to function at low temperatures but may have also arisen via gene duplications that increased protein functional diversity.

## Materials and methods

### SPAdes assembly

The Illumina HiSeq 2000 PE (paired-end, 100 bp, with BioProject ID SRX1959352) reads obtained after sequencing of *Euplotes focardii* macronuclear genome, previously trimmed using the Trimmomatic software (version 0.36)^82^ and checked using the FastQC software (version 0.11.5)^83^, were assembled using the SPAdes algorithm (version 3.10.1)^84^ with the “careful” option and the BayesHammer error correction algorithm^85^. Other parameters were set to default values. Additionally, SPAdes version 3.9, version 3.11.1 and a different set of k-mer lengths were also used to check and identify the best version and configuration of the assembler for these reads.

Possible redundant “chaff” contigs were removed from the assembly, as previously reported^32^, by mapping contigs shorter than 500 bp that had matches to the other contigs with greater than 80% coverage and 90% sequence identity.

### Assembly clean-up and properties

To perform a quality assessment of the obtained assembly from SPAdes, avoiding bacterial contamination, the assembly was further analyzed checking the GC content by the QUAST software (version 4.5)^86^. The contigs having GC content higher than 45%, and a coverage lower than 10, were removed from the assembly using a custom algorithm written in Perl. This GC content percentage threshold was also set on the base of the minimum GC content of the most abundant bacteria in the consortium associated to *E. focardii* (data unpublished). Further bacterial contaminations were analyzed by using BLAST of the assembly versus the Genbank nr database setting bacteria as taxonomy filter, with 80% of hit coverage and 95% sequence identity of the matches. To check the goodness of the assembly, the SPAdes procedure was further repeated after this decontamination step. Currently, Genbank nr database also includes data of the last reported *Euplotes* endosymbiotic bacteria^87,88^.

Moreover, to remove possible contaminant algal sequences, the *Dunaliella salina* genome (used to feed *E. focardii*) was compared to the *E. focardii* macronuclear genome assembly, using blastn and cd-hit-2d software with a threshold of 0.95 (see "Paralog prediction" section for details).

After all the steps of assembling and cleaning, the assembly was evaluated using another custom Perl script providing information about the size, the number of contigs, the number of telomeres, the number of 2, 1, and 0 telomere contigs and the mean length of contigs.

### Gene prediction and frameshifting analysis

The gene prediction procedure on the *E. focardii* macronuclear genome assembly was performed using the AUGUSTUS software (version 3.3.3)^89^ previously trained and tested on a manually curated data set, with no cases of translation frameshifting, from *Euplotes crassus* (data unpublished). The software was run using the following parameters: “–species = euplotes_crassus –UTR = on –alternatives-from-evidence = true –genemodel = complete –codingseq = on”. The results of this prediction were assessed/processed with another Perl script.

Genes predicted were additionally analyzed checking 12 potential frameshifting sites (5’-AAATAA-3’, 5’-AATTAA-3’, 5’-ATTTAA-3’, 5’-TTATAA-3’, 5’-AACTAA-3’, 5’-ATATAA-3’, 5’-GAGTAA-3’, 5’-AAATAG-3’, 5’-GTATAA-3’, 5’-TTATAG-3’, 5’-ATATAG-3’ and 5’-TCCTAA-3’, ranked by abundance), previously detected in *E. crassus*^28^, located at the end of these sequences and comparing them with the *E. focardii* proteome obtained after the Protein Annotation step. Sequences having these potential sites (7023) were checked and selected on the base of sequence length (lower than the related blast top hit length) and BLAST coverage (higher than 80%) versus the best hit obtained from nr reference database. Moreover, the transcript-level quantification of these genes, in comparison with all the others, was estimated using pair-end Illumina transcriptome reads of *E. focardii* and RSEM software package^90^, which allocates the multi-mapping reads on the base of an expectation maximization approach. A two-sample independent *t*-test was used to perform this comparison (*p*-value < 0.05 were considered to be significant).

### Protein annotation

The proteins predicted from the *E. focardii* macronuclear genome assembly were searched and annotated using the OmicsBox software (version 1.4.11)^91,92^. The parameter settings used for the procedure were: blastp as blast program, nr as blastdb, 1.0e^−3^ as E-value and 20 as number of BLAST hits. Other settings for the annotation were set to the default values.

### Assessment of genome completeness

The assessment of genome completeness was firstly conducted analyzing the percentage of conserved core eukaryotic genes (CEGs)^93^ searching the number of protein sequences contained in the CEG database (composed by 248 proteins) that were likely homologs with those of the *E. focardii* macronuclear genome assembly (i.e., with blastp E-values lower than 1e-10 and a match coverage higher than 70% of the length of the CEG proteins).

The tRNAs were initially predicted with the Aragorn algorithm (version 1.2.41)^94^. Ribosomal proteins were counted after the protein annotation, as previously described, and structural RNAs were identified by BLAST searches of the assembly against the Rfam database. Secondary and tertiary structures of a potential stop-suppressor tRNA were determined using the RNAfold web server^95^ and the RNAComposer automated RNA structure modeling server^96^, respectively.

### Paralog prediction

The first step of this analysis was to cluster the *E. focardii* macronuclear proteome using the cd-hit software (version 4.7)^97,98^ with a sequence identity threshold of 0.95 to merge alleles (26,680 clusters). Therefore, the clustering was performed using a threshold of 0.4 to identify the largest and most represented protein families in the proteome (21,850 clusters of which 2312 with at least two elements). In this work, we have focused our interest on the Hsp70, tubulins and antioxidant enzymes family. Moreover, CD-HIT (and CD-HIT-EST) software was also used with a threshold of 0.95 to identify possible alternative fragmentation in the whole genome.

Pairwise sequence identity searches were performed on the *E. focardii* MAC genome assembly, in comparison with the *E. crassus*, *E. octocarinatus* and *E. vannus* assemblies, to estimate the distribution of alleles and paralogous sequences. By a custom Perl script, an alignment of all contigs against each other was performed into the assembly invoking the blastn algorithm and extracting the best non-self BLAST hits; then, MAFFT algorithm (–clustalout –maxiterate 1000 –globalpair / –localpair) was invoked to align the two sequences of each pair obtained; finally, its sequence identity was calculated.

### tRNA sequencing, mapping and quantification

Total RNA was extracted using TRI Reagent (Sigma) according to the manufacturer’s protocol, deacylated at 37 °C for 40 min in 20 mM Tris–HCl (pH 9.0) and precipitated with 5 M ammonium acetate in 75% ethanol. This RNA was used to produce a library of mature tRNAs by the YAMAT-seq method^99^. The resulting cDNA library was multiplexed and sequenced on an Illumina MiSeq sequencer in paired-end mode (75 bp reads).

Paired-end YAMAT-seq reads were merged with BBMerge (default parameters), from the BBTools package^100^. Forward and reverse adapters were trimmed off the merged reads using cutadapt 3.2 (default parameters; -m 20)^101^. This procedure yielded 623,000 reads, which were mapped to *E. focardii* tRNAs predicted by tRNAscan-SE 2.0 as a part of the tRAX pipeline^102^ (default parameters) which was also used to obtain read counts (available as Supplementary Table 1).

### Hsp70, tubulins and antioxidant enzymes system classification

The classes of Hsp70, tubulins and antioxidant enzymes in *E. focardii* previously detected and analyzed^1,69^ were confirmed and extended after the protein annotation and ""Paralog prediction stages. Clustal Omega algorithm^103^ was used to produce each multi-alignment, and MEGA version X^104^ was used to infer each phylogeny using the Maximum Likelihood statistical method, the JTT substitution model with a single substitution rate category (for SODs, it was used with four substitution rate categories), and generate 100 bootstrap pseudo-replicates. MEGA version X^104^ was also used to plot each phylogenetic tree.

### Homology modeling and molecular dynamics of alpha tubulins

Three dimensional structures of α-tubulins of *Euplotes* species, not already available by X-ray crystallographic or NMR analysis, were obtained by homology modeling using the Modeller software (version 9.20)^105^ and the Swiss-Model server^106^. Three dimensional structures (reported as pdb ID) detected as best template to model the α-tubulins of *Euplotes*, on the base of the Global Model Quality Estimation (GMQE) score, the coverage and the sequence identity in the Swiss-Model server, were: 6U0H^10^, used for TBα1 (with 96.20% of identity), TBα2 (94.63%), and TBα3 (83.87%) of *E. focardii*, for TBα1 (93.93%), TBα2 (92.19%), and TBα4 (90.02%) of *E. crassus*, and for TBα1 (95.74%) of *E. octocarinatus*; 6E88^108^, used for TBα4 (52.53%), and TBα7 (49.31%) of *E. focardii*; 1IA0^109^, used for TBα5 (56.06%) of *E. focardii*; 5W3F^110^, used for TBα6 (52.58%) of *E. focardii*; 6U42^111^, used for TBα3 (52.58%) of *E. crassus*; 6U0T^107^, used for TBα5 (52.58%) of *E. crassus*. Final homology models were assessed using the zDOPE score and the estimated RMSD/Overlap in Modeller and the Q-MEAN score in the Swiss-Model server.

Molecular dynamics (MD) was performed using GROMACS (version 2019.3)^112^ and the all-atom OPLS force field. After the preliminary topology generation, macromolecules were soaked in a SPC water molecules cubic box in the presence of Na^+^/Cl^−^ ions. The entire system was minimized, until the variation of potential energy was smaller than 100 kJ mol^−1^ nm^−1^, using the steepest descent algorithm and equilibrated under a constant Number of particles, Volume, and Temperature (NVT) and a constant Number of particles, Pressure and Temperature (NPT) ensemble. Then, the MD simulation was run for 50 ns. Temperatures in the equilibration and MD simulation steps were set either at 4 °C (277 K) or at 27 °C (300 K). The backbone root mean square deviation (RMSD) and the protein root mean square fluctuation (RMSF) were determined using the GROMACS tools “gmx rms” and “gmx rmsf”, respectively, while the maximum RMSD value (*RMSD*_*max*_) for each dynamic was calculated fitting the trajectory with the equation *RMSD* = *t* * *RMSD*_*max*_ / (*t*^*n*^ + *const*) (Fig. 3A).

#### *Hsp70* gene transcription by *E. focardii* under stress

We evaluated the inducibility of *E. focardii Hsp70* genes in cultures subjected to thermal or oxidative stress. Cells entering stationary phase after ~ 1 week of vegetative proliferation in the presence of food (the green alga *Dunaliella tertiolecta*) were pelleted by low-speed centrifugation (500×*g*, 3 min), and pellets were resuspended in seawater to a density of ~ 5 × 10^3^ cells/mL. Heat-shock was performed by warming cells from 4 to 18 °C over 30 min. Control cells were incubated at 4 °C for 30 min. Oxidative stress was produced by incubating cells at 4 °C in the presence of 100 µM H_2_O_2_ for 30 min. Total RNA from control or stressed cells was extracted with Trizol reagent (GIBCO BRL), and cDNA was synthesized from each template using the StrataScript Reverse Transcriptase (Stratagene).

Transcript levels corresponding to the seven *E. focardii Hsp70* genes were measured in control and stressed DNA samples by comparative-threshold qPCR using the SYBR green DNA-binding method^113^ and the primer pairs given in Supplemental Tables 2 and 3; the *Euplotes* SSrDNA gene (GenBank ID**:** EF094961) was used for normalization. To 100 ng of *E. focardii* cDNA were added 12.5 μl of 2 × SYBR Green JumpStart Taq ReadyMix (Sigma-Aldrich, Milan), 5 pg each of gene-specific forward and reverse primers, and water to a final volume of 25 μl. Amplification reactions were performed in triplicate in a Multicolor qPCR MX3000P thermocycler (Stratagene, Milan, Italy), with an initial denaturation step (95 °C for 2 min) to activate the polymerase followed by 45 cycles of denaturation at 95 °C for 30 s, and annealing and extension at 60 °C for 15 s. During annealing/extension, the increase in fluorescence at 495 nm was monitored, and the threshold value was set at 30 units. To verify that the primer pairs gave specific PCR products without non-specific amplification, the DNA samples were subjected to melting curve analysis by ramping the thermocycler temperature from 50 to 95 °C at 0.05 °C/sec.

The relative expression of the *Hsp70* genes was calculated by the method of Pfaffl^114^:

$$Relative\;expression\;ratio = \, \left( {E_{{{\text{target}}}} } \right)^{{\Delta {\text{Ct}}}}_{{{\text{target}}(control - sample)}} / \, \left( {E_{{{\text{ref}}}} } \right)^{{\Delta {\text{Ct}}}}_{{{\text{ref}}(control - sample)}}$$

where Ct is the PCR cycle number at which the fluorescent signal is above the set threshold, ∆Ct is the Ct difference (control minus sample) of the target or reference gene, and *E* is the real-time PCR efficiency of the target or reference gene (*E* = 10^–1/slope^, calculated from plots of Ct *vs.* cDNA input). The relative expression ratios of transcripts under investigation were tested for statistical significance by a pairwise, fixed reallocation randomization test implemented in REST MCS version 2 software^115^.

## Supplementary Information

Supplementary Information.

## References

[1] Pucciarelli S (2009). Molecular cold-adaptation of protein function and gene regulation: the case for comparative genomic analyses in marine ciliated protozoa. Mar Genomics.

[2] Pucciarelli S, Marziale F, Di Giuseppe G, Barchetta S, Miceli C (2005). Ribosomal cold-adaptation: characterization of the genes encoding the acidic ribosomal P0 and P2 proteins from the Antarctic ciliate Euplotes focardii. Gene.

[3] Pucciarelli S, Miceli C (2002). Characterization of the cold-adapted alpha-tubulin from the psychrophilic ciliate Euplotes focardii. Extremophiles.

[4] Yang G (2013). Characterization and comparative analysis of psychrophilic and mesophilic alpha-amylases from Euplotes species: a contribution to the understanding of enzyme thermal adaptation. Biochem Biophys Res Commun.

[5] Prescott DM (1994). The DNA of ciliated protozoa. Microbiol Rev.

[6] Mollenbeck M, Klobutcher LA (2002). De novo telomere addition to spacer sequences prior to their developmental degradation in Euplotes crassus. Nucleic Acids Res.

[7] Swart EC (2013). The Oxytricha trifallax macronuclear genome: a complex eukaryotic genome with 16,000 tiny chromosomes. PLoS Biol.

[8] Heyse G, Jonsson F, Chang WJ, Lipps HJ (2010). RNA-dependent control of gene amplification. Proc Natl Acad Sci U S A.

[9] Nowacki M, Haye JE, Fang W, Vijayan V, Landweber LF (2010). RNA-mediated epigenetic regulation of DNA copy number. Proc Natl Acad Sci U S A.

[10] Dayeh VR (2005). Comparing a ciliate and a fish cell line for their sensitivity to several classes of toxicants by the novel application of multiwell filter plates to Tetrahymena. Res Microbiol.

[11] Detrich, H. W., 3rd, Parker, S. K., Williams, R. C., Jr., Nogales, E. & Downing, K. H. Cold adaptation of microtubule assembly and dynamics. Structural interpretation of primary sequence changes present in the alpha- and beta-tubulins of Antarctic fishes. *J Biol Chem* **275**, 37038–37047. 10.1074/jbc.M005699200 (2000). 10.1074/jbc.M005699200 10956651

[12] Manka SW, Moores CA (2018). Microtubule structure by cryo-EM: snapshots of dynamic instability. Essays Biochem.

[13] Inclan YF, Nogales E (2001). Structural models for the self-assembly and microtubule interactions of gamma-, delta- and epsilon-tubulin. J Cell Sci.

[14] Chiappori F (2012). Structural thermal adaptation of beta-tubulins from the Antarctic psychrophilic protozoan Euplotes focardii. Proteins.

[15] Marziale F (2008). Different roles of two gamma-tubulin isotypes in the cytoskeleton of the Antarctic ciliate Euplotes focardii: remodelling of interaction surfaces may enhance microtubule nucleation at low temperature. FEBS J.

[16] Pucciarelli S, Miceli C, Melki R (2002). Heterologous expression and folding analysis of a beta-tubulin isotype from the Antarctic ciliate Euplotes focardii. Eur J Biochem.

[17] Gromer S, Urig S, Becker K (2004). The thioredoxin system–from science to clinic. Med Res Rev.

[18] Birben E, Sahiner UM, Sackesen C, Erzurum S, Kalayci O (2012). Oxidative stress and antioxidant defense. World Allergy Organ J.

[19] Alin P, Danielson UH, Mannervik B (1985). 4-Hydroxyalk-2-enals are substrates for glutathione transferase. FEBS Lett.

[20] Juganson K (2017). Mechanisms of toxic action of silver nanoparticles in the protozoan Tetrahymena thermophila: From gene expression to phenotypic events. Environ Pollut.

[21] Clark MS, Fraser KP, Peck LS (2008). Antarctic marine molluscs do have an HSP70 heat shock response. Cell Stress Chaperones.

[22] Tomanek L (2002). The heat-shock response: its variation, regulation and ecological importance in intertidal gastropods (genus Tegula). Integr Comp Biol.

[23] Morimoto RI, Kline MP, Bimston DN, Cotto JJ (1997). The heat-shock response: regulation and function of heat-shock proteins and molecular chaperones. Essays Biochem.

[24] Gonzalez-Aravena M (2018). HSP70 from the Antarctic sea urchin Sterechinus neumayeri: molecular characterization and expression in response to heat stress. Biol Res.

[25] Hofmann GE, Buckley BA, Airaksinen S, Keen JE, Somero GN (2000). Heat-shock protein expression is absent in the antarctic fish Trematomus bernacchii (family Nototheniidae). J Exp Biol.

[26] La Terza, A., Papa, G., Miceli, C. & Luporini, P. Divergence between two Antarctic species of the ciliate Euplotes, E. focardii and E. nobilii, in the expression of heat-shock protein 70 genes. *Mol Ecol* **10**, 1061–1067. 10.1046/j.1365-294x.2001.01242.x (2001). 10.1046/j.1365-294x.2001.01242.x 11348511

[27] Klobutcher LA, Farabaugh PJ (2002). Shifty ciliates: frequent programmed translational frameshifting in euplotids. Cell.

[28] Lobanov AV (2017). Position-dependent termination and widespread obligatory frameshifting in Euplotes translation. Nat Struct Mol Biol.

[29] Coordinators NR (2017). Database resources of the National Center for Biotechnology Information. Nucleic Acids Res.

[30] Pucciarelli S (2015). Microbial consortium associated with the antarctic marine ciliate Euplotes focardii: an investigation from genomic sequences. Microb Ecol.

[31] Klobutcher LA (1998). Conserved DNA sequences adjacent to chromosome fragmentation and telomere addition sites in Euplotes crassus. Nucleic Acids Res.

[32] Aeschlimann SH (2014). The draft assembly of the radically organized Stylonychia lemnae macronuclear genome. Genome Biol Evol.

[33] Swart, E. C. (personal communication).

[34] Cavalcanti AR, Stover NA, Orecchia L, Doak TG, Landweber LF (2004). Coding properties of Oxytricha trifallax (Sterkiella histriomuscorum) macronuclear chromosomes: analysis of a pilot genome project. Chromosoma.

[35] Lozupone CA, Knight RD, Landweber LF (2001). The molecular basis of nuclear genetic code change in ciliates. Curr Biol.

[36] Salas-Marco J (2006). Distinct paths to stop codon reassignment by the variant-code organisms Tetrahymena and Euplotes. Mol Cell Biol.

[37] Klobutcher LA (2005). Sequencing of random Euplotes crassus macronuclear genes supports a high frequency of +1 translational frameshifting. Eukaryot Cell.

[38] Wang R, Xiong J, Wang W, Miao W, Liang A (2016). High frequency of +1 programmed ribosomal frameshifting in Euplotes octocarinatus. Sci Rep.

[39] Turanov AA (2009). Genetic code supports targeted insertion of two amino acids by one codon. Science.

[40] Maehigashi T, Dunkle JA, Miles SJ, Dunham CM (2014). Structural insights into +1 frameshifting promoted by expanded or modification-deficient anticodon stem loops. Proc Natl Acad Sci U S A.

[41] Miceli C, Ballarini P, Di Giuseppe G, Valbonesi A, Luporini P (1994). Identification of the tubulin gene family and sequence determination of one beta-tubulin gene in a cold-poikilotherm protozoan, the antarctic ciliate Euplotes focardii. J Eukaryot Microbiol.

[42] Ricci F (2019). The sub-chromosomic macronuclear pheromone genes of the ciliate Euplotes raikovi: comparative structural analysis and insights into the mechanism of expression. J Eukaryot Microbiol.

[43] Wang R, Liu J, Di Giuseppe G, Liang A (2020). UAA and UAG may Encode Amino Acid in Cathepsin B Gene of Euplotes octocarinatus. J Eukaryot Microbiol.

[44] Heaphy SM, Mariotti M, Gladyshev VN, Atkins JF, Baranov PV (2016). Novel ciliate genetic code variants including the reassignment of all three stop codons to sense codons in condylostoma magnum. Mol Biol Evol.

[45] Swart EC, Serra V, Petroni G, Nowacki M (2016). Genetic codes with no dedicated stop codon: context-dependent translation termination. Cell.

[46] Roy B, Leszyk JD, Mangus DA, Jacobson A (2015). Nonsense suppression by near-cognate tRNAs employs alternative base pairing at codon positions 1 and 3. Proc Natl Acad Sci U S A.

[47] Dunn JG, Foo CK, Belletier NG, Gavis ER, Weissman JS (2013). Ribosome profiling reveals pervasive and regulated stop codon readthrough in Drosophila melanogaster. Elife.

[48] Frechin M, Duchene AM, Becker HD (2009). Translating organellar glutamine codons: a case by case scenario?. RNA Biol.

[49] Wilcox M, Nirenberg M (1968). Transfer RNA as a cofactor coupling amino acid synthesis with that of protein. Proc Natl Acad Sci U S A.

[50] Detrich HW, Fitzgerald TJ, Dinsmore JH, Marchese-Ragona SP (1992). Brain and egg tubulins from antarctic fishes are functionally and structurally distinct. J Biol Chem.

[51] Detrich HW, Johnson KA, Marchese-Ragona SP (1989). Polymerization of Antarctic fish tubulins at low temperatures: energetic aspects. Biochemistry.

[52] Wloga D (2008). Glutamylation on alpha-tubulin is not essential but affects the assembly and functions of a subset of microtubules in Tetrahymena thermophila. Eukaryot Cell.

[53] Eisen JA (2006). Macronuclear genome sequence of the ciliate Tetrahymena thermophila, a model eukaryote. PLoS Biol.

[54] Aury JM (2006). Global trends of whole-genome duplications revealed by the ciliate Paramecium tetraurelia. Nature.

[55] Pucciarelli S (2012). Distinct functional roles of beta-tubulin isotypes in microtubule arrays of Tetrahymena thermophila, a model single-celled organism. PLoS ONE.

[56] Pucciarelli S (2013). Tubulin folding: the special case of a beta-tubulin isotype from the Antarctic psychrophilic ciliate Euplotes focardii. Polar Biol.

[57] Pucci F, Rooman M (2017). Physical and molecular bases of protein thermal stability and cold adaptation. Curr Opin Struct Biol.

[58] Aqvist J, Isaksen GV, Brandsdal BO (2017). Computation of enzyme cold adaptation. Nat Rev Chem.

[59] Lesser MP (2006). Oxidative stress in marine environments: biochemistry and physiological ecology. Annu Rev Physiol.

[60] McCord, J. M. & Fridovich, I. Superoxide dismutase. An enzymic function for erythrocuprein (hemocuprein). *J Biol Chem* **244**, 6049–6055 (1969). 5389100

[61] McCord JM, Fridovich I (1988). Superoxide dismutase: the first twenty years (1968–1988). Free Radic Biol Med.

[62] Miller AF (2012). Superoxide dismutases: ancient enzymes and new insights. FEBS Lett.

[63] Benov LT, Fridovich I (1994). Escherichia coli expresses a copper- and zinc-containing superoxide dismutase. J Biol Chem.

[64] Steinman HM, Ely B (1990). Copper-zinc superoxide dismutase of Caulobacter crescentus: cloning, sequencing, and mapping of the gene and periplasmic location of the enzyme. J Bacteriol.

[65] Antonyuk, S. V., Strange, R. W., Marklund, S. L. & Hasnain, S. S. The structure of human extracellular copper-zinc superoxide dismutase at 1.7 A resolution: insights into heparin and collagen binding. *J Mol Biol* **388**, 310–326. 10.1016/j.jmb.2009.03.026 (2009). 10.1016/j.jmb.2009.03.026 19289127

[66] Marklund SL (1984). Extracellular superoxide dismutase and other superoxide dismutase isoenzymes in tissues from nine mammalian species. Biochem J.

[67] Bannister JV, Bannister WH, Rotilio G (1987). Aspects of the structure, function, and applications of superoxide dismutase. CRC Crit Rev Biochem.

[68] James ER (1994). Superoxide dismutase. Parasitol Today.

[69] Ferro D (2015). Cu, Zn superoxide dismutases from Tetrahymena thermophila: molecular evolution and gene expression of the first line of antioxidant defenses. Protist.

[70] Arnaiz O, Sperling L (2011). ParameciumDB in 2011: new tools and new data for functional and comparative genomics of the model ciliate Paramecium tetraurelia. Nucleic Acids Res.

[71] Fink RC, Scandalios JG (2002). Molecular evolution and structure–function relationships of the superoxide dismutase gene families in angiosperms and their relationship to other eukaryotic and prokaryotic superoxide dismutases. Arch Biochem Biophys.

[72] Lee YM, Friedman DJ, Ayala FJ (1985). Superoxide dismutase: an evolutionary puzzle. Proc Natl Acad Sci U S A.

[73] Pischedda A (2018). Antarctic marine ciliates under stress: superoxide dismutases from the psychrophilic Euplotes focardii are cold-active yet heat tolerant enzymes. Sci Rep.

[74] Yang G (2013). Characterization of the first eukaryotic cold-adapted patatin-like phospholipase from the psychrophilic Euplotes focardii: Identification of putative determinants of thermal-adaptation by comparison with the homologous protein from the mesophilic Euplotes crassus. Biochimie.

[75] Li J, Zhou L, Lin X, Yi Z, Al-Rasheid KA (2014). Characterizing dose-responses of catalase to nitrofurazone exposure in model ciliated protozoan Euplotes vannus for ecotoxicity assessment: enzyme activity and mRNA expression. Ecotoxicol Environ Saf.

[76] Prast-Nielsen S, Huang HH, Williams DL (1810). Thioredoxin glutathione reductase: its role in redox biology and potential as a target for drugs against neglected diseases. Biochim Biophys Acta.

[77] Kabani M, Martineau CN (2008). Multiple hsp70 isoforms in the eukaryotic cytosol: mere redundancy or functional specificity?. Curr Genomics.

[78] La Terza A, Miceli C, Luporini P (2004). The gene for the heat-shock protein 70 of Euplotes focardii, an Antarctic psychrophilic ciliate. Antarct. Sci..

[79] Chen X (2019). Genome analyses of the new model protist Euplotes vannus focusing on genome rearrangement and resistance to environmental stressors. Mol Ecol Resour.

[80] Chen Z (2008). Transcriptomic and genomic evolution under constant cold in Antarctic notothenioid fish. Proc Natl Acad Sci U S A.

[81] Li Y (2019). Comparative transcriptomic analysis reveals gene expression associated with cold adaptation in the tea plant Camellia sinensis. BMC Genomics.

[82] Bolger AM, Lohse M, Usadel B (2014). Trimmomatic: a flexible trimmer for Illumina sequence data. Bioinformatics.

[83] Andrews, S. (2010).

[84] Bankevich A (2012). SPAdes: a new genome assembly algorithm and its applications to single-cell sequencing. J Comput Biol.

[85] Nikolenko, S. I., Korobeynikov, A. I. & Alekseyev, M. A. BayesHammer: Bayesian clustering for error correction in single-cell sequencing. *BMC Genomics* **14 Suppl 1**, S7. 10.1186/1471-2164-14-S1-S7 (2013). 10.1186/1471-2164-14-S1-S7 PMC3549815 23368723

[86] Gurevich A, Saveliev V, Vyahhi N, Tesler G (2013). QUAST: quality assessment tool for genome assemblies. Bioinformatics.

[87] Boscaro V, Husnik F, Vannini C, Keeling PJ (2019). Symbionts of the ciliate Euplotes: diversity, patterns and potential as models for bacteria-eukaryote endosymbioses. Proc Biol Sci.

[88] Serra, V. *et al.* Morphology, ultrastructure, genomics, and phylogeny of Euplotes vanleeuwenhoeki sp. nov. and its ultra-reduced endosymbiont "Candidatus Pinguicoccus supinus" sp. nov. *Sci Rep* **10**, 20311. 10.1038/s41598-020-76348-z (2020). 10.1038/s41598-020-76348-z PMC7679464 33219271

[89] Stanke M (2006). AUGUSTUS: ab initio prediction of alternative transcripts. Nucleic Acids Res.

[90] Li B, Dewey CN (2011). RSEM: accurate transcript quantification from RNA-Seq data with or without a reference genome. BMC Bioinformatics.

[91] Conesa A (2005). Blast2GO: a universal tool for annotation, visualization and analysis in functional genomics research. Bioinformatics.

[92] Gotz S (2008). High-throughput functional annotation and data mining with the Blast2GO suite. Nucleic Acids Res.

[93] Parra G, Bradnam K, Korf I (2007). CEGMA: a pipeline to accurately annotate core genes in eukaryotic genomes. Bioinformatics.

[94] Laslett D, Canback B (2004). ARAGORN, a program to detect tRNA genes and tmRNA genes in nucleotide sequences. Nucleic Acids Res.

[95] Gruber AR, Lorenz R, Bernhart SH, Neubock R, Hofacker IL (2008). The Vienna RNA websuite. Nucleic Acids Res.

[96] Popenda M (2012). Automated 3D structure composition for large RNAs. Nucleic Acids Res.

[97] Fu L, Niu B, Zhu Z, Wu S, Li W (2012). CD-HIT: accelerated for clustering the next-generation sequencing data. Bioinformatics.

[98] Li W, Godzik A (2006). Cd-hit: a fast program for clustering and comparing large sets of protein or nucleotide sequences. Bioinformatics.

[99] Shigematsu M (2017). YAMAT-seq: an efficient method for high-throughput sequencing of mature transfer RNAs. Nucleic Acids Res.

[100] Bushnell B, Rood J, Singer E (2017). BBMerge: accurate paired shotgun read merging via overlap. PLoS ONE.

[101] Martin, M. Cutadapt removes adapter sequences from high-throughput sequencing reads. *2011* **17**, 3. 10.14806/ej.17.1.200 (2011).

[102] Holmes, A. D., Howard, J. M., Chan, P. P. & Lowe, T. M. tRNA Analysis of eXpression (tRAX): A tool for integrating analysis of tRNAs, tRNA-derived small RNAs, and tRNA modifications. *(Submitted)* (2020).

[103] Sievers, F. & Higgins, D. G. Clustal omega. *Curr Protoc Bioinformatics* **48**, 3 13 11–16. 10.1002/0471250953.bi0313s48 (2014). 10.1002/0471250953.bi0313s48 25501942

[104] Kumar S, Stecher G, Li M, Knyaz C, Tamura K (2018). MEGA X: molecular evolutionary genetics analysis across computing platforms. Mol Biol Evol.

[105] Webb, B. & Sali, A. Comparative protein structure modeling using MODELLER. *Curr Protoc Bioinform.* **54**, 5 6 1–5 6 37. 10.1002/cpbi.3 (2016). 10.1002/cpbi.3 PMC5031415 27322406

[106] Waterhouse A (2018). SWISS-MODEL: homology modelling of protein structures and complexes. Nucleic Acids Res.

[107] Ichikawa M (2019). Tubulin lattice in cilia is in a stressed form regulated by microtubule inner proteins. Proc Natl Acad Sci U S A.

[108] Chaaban, S. *et al.* The Structure and Dynamics of C. elegans Tubulin Reveals the Mechanistic Basis of Microtubule Growth. *Dev Cell* **47**, 191–204 e198. 10.1016/j.devcel.2018.08.023 (2018). 10.1016/j.devcel.2018.08.023 30245157

[109] Kikkawa M (2001). Switch-based mechanism of kinesin motors. Nature.

[110] Howes SC (2017). Structural differences between yeast and mammalian microtubules revealed by cryo-EM. J Cell Biol.

[111] Ma, M. *et al.* Structure of the Decorated Ciliary Doublet Microtubule. *Cell* **179**, 909–922 e912. 10.1016/j.cell.2019.09.030 (2019). 10.1016/j.cell.2019.09.030 PMC6936269 31668805

[112] Abraham MJ (2015). GROMACS: High performance molecular simulations through multi-level parallelism from laptops to supercomputers. SoftwareX.

[113] Morrison, T. B., Weis, J. J. & Wittwer, C. T. Quantification of low-copy transcripts by continuous SYBR Green I monitoring during amplification. *Biotechniques* **24**, 954–958, 960, 962 (1998). 9631186

[114] Pfaffl MW (2001). A new mathematical model for relative quantification in real-time RT-PCR. Nucleic Acids Res.

[115] Pfaffl MW, Horgan GW, Dempfle L (2002). Relative expression software tool (REST) for group-wise comparison and statistical analysis of relative expression results in real-time PCR. Nucleic Acids Res.

