# Supplementary material for: The macronuclear genome of the Antarctic psychrophilic marine ciliate Euplotes focardii reveals new insights on molecular cold adaptation
Source: Sci Rep. 2021 Sep 21;11:18782. doi: 10.1038/s41598-021-98168-5 (PMC8455672; doi:10.1038/s41598-021-98168-5)
Supplement: Supplementary file 1 — Supplementary Information. [file 41598_2021_98168_MOESM1_ESM.docx]

**Supplementary figure 1** – GC content versus coverage of telomere contigs in red dots (A), of non-telomere contigs in red dots (B) and of contigs in the final cleaned assembly in green dots (C) in comparison with the total contigs obtained by the SPAdes assembler (blue dots).

**
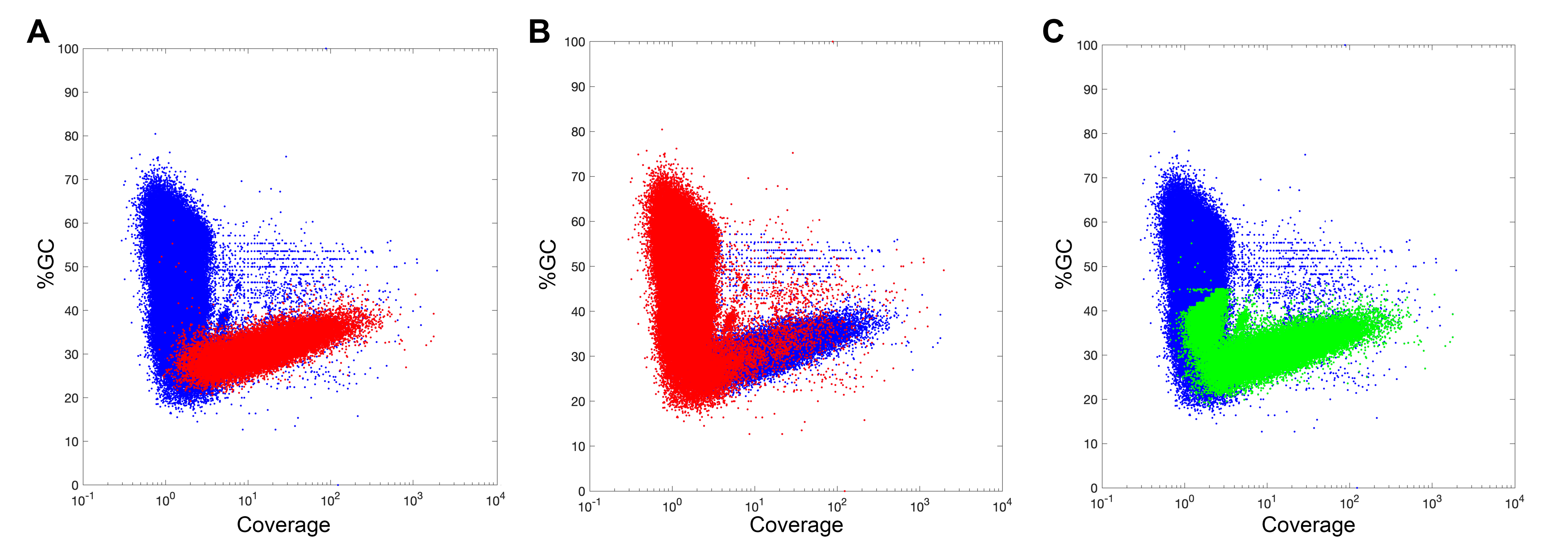
**

**Supplementary figure 2** – GC content distribution of number of contigs in the first, not cleaned, assembly (green), of telomere contigs (blue), of non-telomere contigs (orange), and of contigs in the final cleaned assembly (red).

**
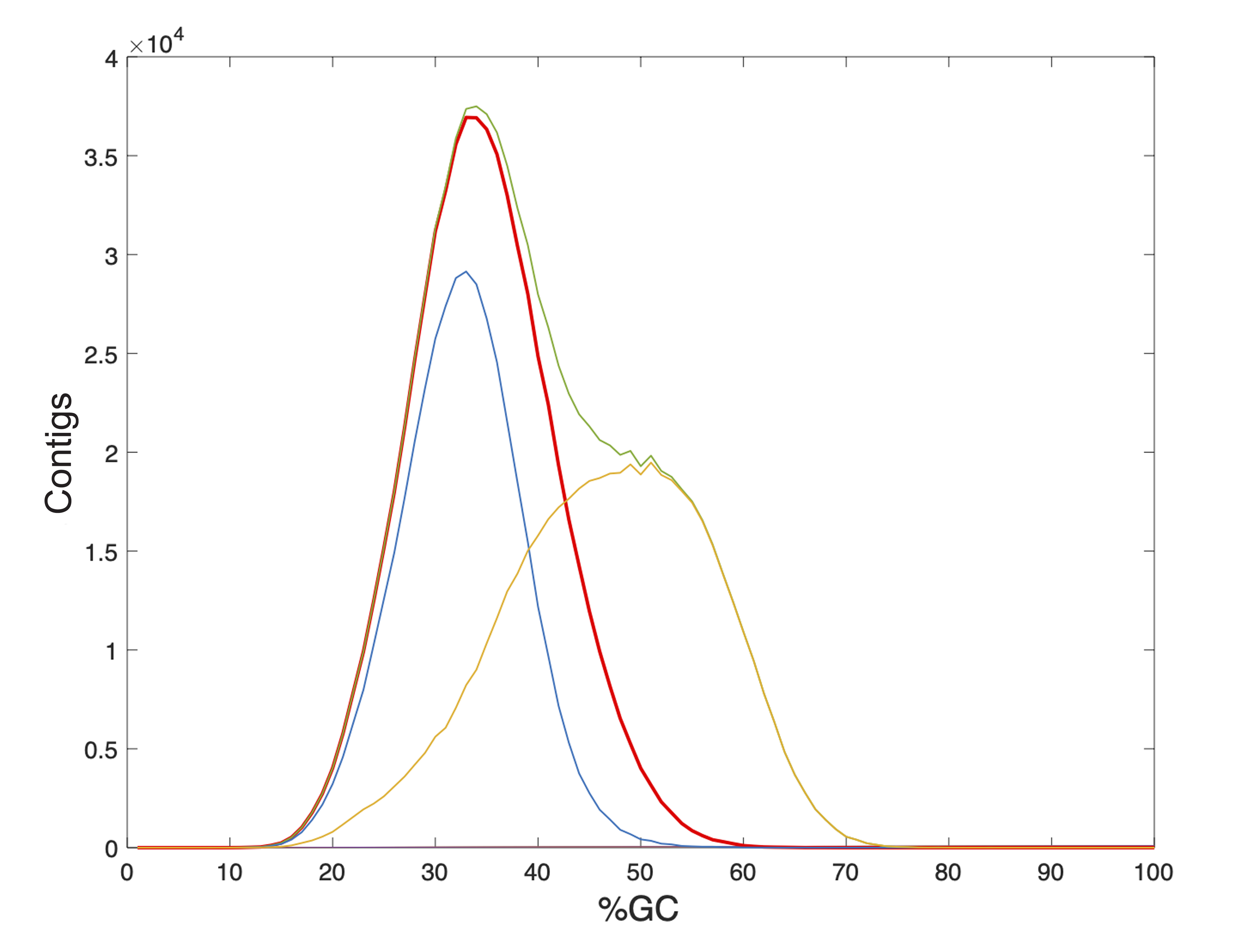
**

**Supplementary figure 3** – Distribution of the pairwise sequence identity between complete nanochromosomes of the *E. focardii*, *E. crassus*, *E. octocarinatus* and *E. vannus* assembly.

**
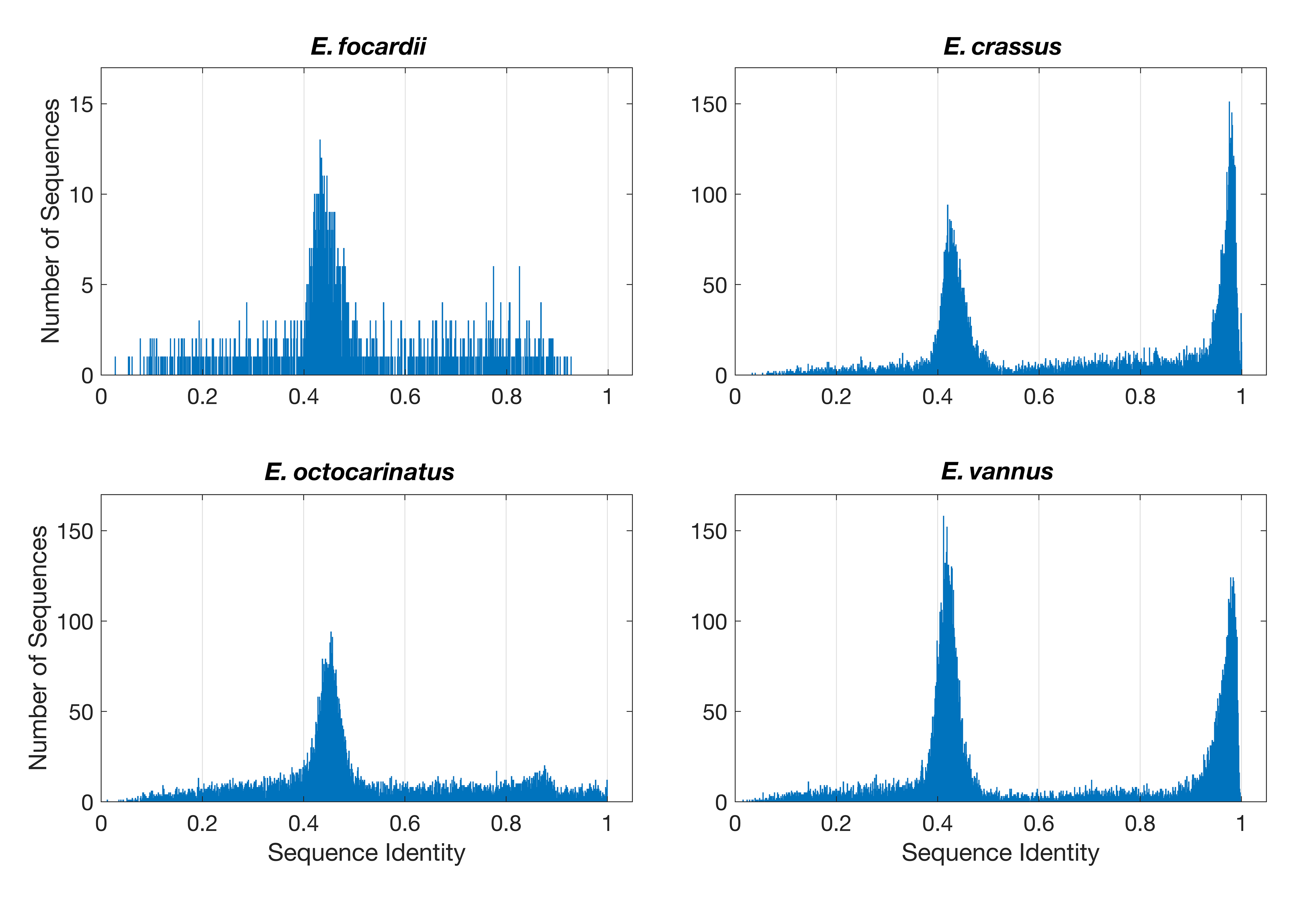
**

**Supplementary table I** – tRNA expression from YAMAT-seq.

| **tRNA type (with NODE ID)** | **Counts from tRAX** |
| --- | --- |
| tRNA-Ala-GGC-NODE_19031-1 | 9 |
| tRNA-Ala-GGC-NODE_38007-1 | 10 |
| tRNA-Ala-TGC-NODE_53974-1 | 51 |
| tRNA-Asn-GTT-NODE_1235-1 | 106 |
| tRNA-Asp-GTC-NODE_41900-1 | 7 |
| tRNA-Gln-CTG-NODE_55663-1 | 33 |
| tRNA-Gln-TTG-NODE_55466-1 | 80 |
| tRNA-Gly-CCC-NODE_58515-1 | 5 |
| tRNA-Gly-GCC-NODE_29499-1 | 5 |
| tRNA-Gly-TCC-NODE_37097-1 | 6 |
| tRNA-Pro-TGG-NODE_57086-1 | 26 |
| tRNA-Ala-AGC-NODE_54234 | 64 |
| tRNA-Ala-CGC-NODE_84835 | 402 |
| tRNA-Ala-GGC-NODE_19031 | 1990 |
| tRNA-Ala-GGC-NODE_38007 | 984 |
| tRNA-Ala-TGC-NODE_53974 | 42687 |
| tRNA-Arg-CCG-NODE_46733 | 25 |
| tRNA-Arg-CCT-NODE_44523 | 6 |
| tRNA-Arg-TCT-NODE_55463 | 19 |
| tRNA-Asn-GTT-NODE_17644 | 4502 |
| tRNA-Asn-GTT-NODE_43321 | 19 |
| tRNA-Asp-GTC-NODE_41900 | 16354 |
| tRNA-Gln-CTG-NODE_55663 | 1412 |
| tRNA-Gln-TTG-NODE_55466 | 2927 |
| tRNA-Glu-CTC-NODE_57592 | 7 |
| tRNA-Glu-TTC-NODE_32101 | 8 |
| tRNA-Glu-TTC-NODE_54875 | 1943 |
| tRNA-Gly-ACC-NODE_63788 | 492 |
| tRNA-Gly-CCC-NODE_58515 | 5212 |
| tRNA-Gly-GCC-NODE_29499 | 10133 |
| tRNA-Gly-TCC-NODE_37097 | 6384 |
| tRNA-Gly-TCC-NODE_58205 | 1876 |
| tRNA-His-GTG-NODE_41121 | 143 |
| tRNA-Ile-AAT-NODE_60270 | 7 |
| tRNA-Ile-TAT-NODE_46453 | 12 |
| tRNA-Ile-TAT-NODE_9798 | 12 |
| tRNA-Leu-AAG-NODE_29499 | 62 |
| tRNA-Leu-TAA-NODE_58802 | 11 |
| tRNA-Leu-TAG-NODE_56494 | 29 |
| tRNA-Lys-CTT-NODE_53441 | 46 |
| tRNA-Lys-TTT-NODE_41650 | 161 |
| tRNA-Met-CAT-NODE_37524 | 853 |
| tRNA-Phe-GAA-NODE_1138 | 8 |
| tRNA-Phe-GAA-NODE_46278 | 10 |
| tRNA-Pro-AGG-NODE_59803 | 144 |
| tRNA-Pro-TGG-NODE_57086 | 14291 |
| tRNA-SeC-TCA-NODE_51680 | 1011 |
| tRNA-Ser-AGA-NODE_58917 | 10 |
| tRNA-Ser-GCT-NODE_54695 | 383 |
| tRNA-Ser-TGA-NODE_60375 | 673 |
| tRNA-Sup-CTA-NODE_32101 | 41 |
| tRNA-Thr-TGT-NODE_46659 | 124 |
| tRNA-Thr-TGT-NODE_61534 | 113 |
| tRNA-Trp-CCA-NODE_60494 | 15 |
| tRNA-Val-AAC-NODE_56290 | 68 |
| tRNA-Val-CAC-NODE_58808 | 140 |
| tRNA-Val-TAC-NODE_60037 | 462 |

**Supplementary table II** - Primers used for qRT-PCR to estimate Hsp70 genes expression.

| ***Name of primer*** | ***Tm°*** | ***Sequence*** | ***Protein assembly code*** |
| --- | --- | --- | --- |
| **NODE_7641_Fw** | 62 | GAAGCCACTGATGAAACCAA | Protein_07400 |
| **NODE_7641_Rev** | 62 | TCCTTCGAATGCTTTGATTG |  |
| **NODE_5582_Fw** | 62 | GCTCTCTTCTTAGCCTTGCTTT | Protein_05367 |
| **NODE_5582_Rev** | 62 | GAAGTGGAATCCTCTCTGTCAA |  |
| **NODE_6610_Fw** | 63 | GCACCTCTTTCCACAGGAAT | Protein_06381 |
| **NODE_6610_Rev** | 62 | GGTTGGTTATCGGCGTAAGT |  |
| **NODE_8261_Fw** | 66 | ATGGCCCCAAGAGGACACCC | Protein_07970 |
| **NODE_8261_Rev** | 58 | TCGGCTTCATTCTTGATCTC |  |
| **NODE_11344_Fw** | 60 | TCGCATCCACCACTTGAAGG | Protein_10662 |
| **NODE_11344_Rev** | 60 | CTCGAGCCTGAAGGAGCATC |  |
| **NODE_4842_Fw** | 59 | TCGAGGTCGACTTGGAAACC | Protein_04607 |
| **NODE_4842_Rev** | 59 | TCCAAAGTGGCTCTAACCCAT |  |
| **NODE_5436_Fw** | 60 | TCATTTGGGCGACAGCTGAA | Protein_05210 |
| **NODE_5436_Rev** | 59 | CCTGTCTGGCAACCTAGTCA |  |

**Supplementary table III** - Hsp70 protein family identification

| ***Protein assembly code*** | **COOH terminal domain sequence** | **Hsp70 family** |
| --- | --- | --- |
| Protein_06381 | DELD | canonical cytoplasmic |
| Protein_05210 | KEES | Non-canonical cytoplasmic |
| Protein_07970 | QKKE | mitochondrial |
| Protein_04607 | KEDL | ribosomal |
| Protein_05367 | DLEL | ribosomal |
| Protein_07400 | HDDL | endoplasmic reticulum |
| Protein_10662 | FESS | endoplasmic reticulum |
